# Heat transfer and hybrid ferrofluid flow over a nonlinearly stretchable rotating disk under the influence of an alternating magnetic field

**DOI:** 10.1038/s41598-022-21784-2

**Published:** 2022-10-20

**Authors:** Abdul Rauf, Aqsa Mushtaq, Nehad Ali Shah, Thongchai Botmart

**Affiliations:** 1 Department of Mathematics, Air University Multan Campus, Chak 5-Faiz, Bahawalpur Road, Multan, Pakistan; 2 Department of Mechanical Engineering, Sejong University, Seoul, 05006 South Korea; 3 Department of Mathematics, Faculty of Science, Khon Kaen University, Khon Kaen, 40002 Thailand

**Keywords:** Mathematics and computing, Nanoscience and technology, Nanoscale materials

## Abstract

Under the influence of an alternating magnetic field, flow and heat transfer of a ferrofluid flow over a flexible revolving disc are examined. The flow is hampered by the external magnetic field, which is dependent on the alternating magnetic field's frequency. The current work examines the heat transfer and three-dimensional flow of fluid with high viscosity on a spinning disc that is stretched in a radial direction. The governing equations' symmetries are computed using Lie group theory. In the problem, there is a resemblance that can accomplish with radially stretching velocities divided into two categories, specifically, linear and power-law, by imposing limits from the boundary conditions. The literature has already covered linear stretching, but this is the first discussion of power-law stretching. The governing partial differential is turned into an ordinary differential equations system using additional similarity transformations, which are then numerically handled. The results are presented for hybrid alumina–copper/ethylene glycol (${\text{Al}}_{2} {\text{O}}_{3} - {\text{Cu}}/{\text{EG}}$) nanofluid. The calculated findings are novel, and it has been seen that they accord quite well with those of the earlier extended literature. It has been found that hybrid nanofluid flow outperforms nanofluid flow in terms of Nusselt number or heat transfer rate. The heat transmission in the fluid is reduced as the Prandtl number is increased. The heat transfer increases as dimensionless magnetic field intensity $\xi$ increases. Also, axial velocity and radial velocity decrease as magnetic field intensity increases. As the ferromagnetic interaction parameter is raised, the efficiency of heat transmission decreased. For non-linear stretching with stretching parameter 0 < m < 1, the velocity decreases with the increase in m.

## Introduction

Numerous applications of the study of the flow field caused by a revolving disc have been identified in numerous technical and industrial domains. Fans, turbines, centrifugal pumps, rotors, viscometers, spinning disc reactors, and other rotating bodies are only a few examples of real-world applications for disc rotation. The study of an incompressible viscous fluid across an infinite plane disc rotating with a uniform rotational velocity was first introduced in the renowned article by Von Karman^1^, which established the history of rotating disc flows. Numerous researchers are continuing to look at this model to produce analytical and numerical results for a better understanding of the fluid behaviour caused by rotating discs. Von Karman^1^ first proposed the use of similarity transformations to change the governing Navier Stokes equations for axisymmetric flow into a set of linked nonlinear ordinary differential equations, and Cochran^2^ then reported the numerical findings for these equations. The effects of heat transport over a revolving disc at a constant temperature were examined by Millsaps and Pohlhausen^3^. For large Prandtl numbers, Awad^4^ provided an asymptotic model to investigate the heat transport phenomena over a spinning disc. The flow caused by stretched surfaces finds significant use in the manufacturing sector, particularly in the extrusion of metals and polymers^5–7^. Crane^8^ provided the precise analytic solution for the steady linear stretching of a surface. This issue was expanded to include three dimensions by Wang^9^. Using the Homotopy Analysis Method, Rashidi and Pour^10^ discovered approximative analytical solutions for the flow and heat transmission over a stretched sheet. Fang^11^ was the first to suggest the steady flow over spinning and stretching disk. Recent research on the flow between two extending discs was conducted by Fang and Zhang^12^. More recently, Turkyilmazoglu^13^ examined the combined effects of magnetohydrodynamics on radially stretched discs. We note that the linear radial stretching velocities were the focus of all of this research. Stretching of the sheet may not always be linear in practical circumstances, according to Gupta and Gupta^14^.

By incorporating nanoparticles into the carrier liquid, heat transfer coefficients can be improved^15^. CuO nanofluid with water and ethylene boiling properties have been investigated^16^. For heat transfer applications, the nanofluid's liquid medium is crucial^17^. For nanofluid flow boiling heat transfer, a novel model has been devised^18^. A growing proportion of nanotechnology for heat transmission is called nanofluids, which are colloidal mixtures of nanoparticles (1–100 nm) and base liquid (nanoparticle fluid suspensions). Nanofluids' heat transfer capabilities have been investigated to use them as a coolant^19^. The analysis of heat transmission has been done on multi-wall nanotube nanofluids^20^. Some recent work on nanofluid flow can be seen in^21,22^. A spinning disc created viscous fluid flow, which has been explored by Cochran^2^. Similar issues have been investigated by Benton using recurrence relation approaches^23^. These sorts of difficulties have been expanded for magnetohydrodynamic flow due to their employment in a spinning system^13,24–26^. Due to the technical uses of ferrofluid in a revolving system, research investigation on ferrohydrodynamic flow caused by a spinning disc was carried out. An analytical solution was used to investigate the influence of the viscosity of ferrofluid flow affected by the magnetic field owing to a spinning disc^27^. For ferrohydrodynamic flow in a revolving system, heat transfer analysis and mathematical modeling have been published^28^. It looked at how magnetic field-dependent viscosity affected ferrofluid flow that isn't consistent over a spinning disc^29^.

Magnetic nanoparticles are suspended colloidally in a carrier liquid to form ferrofluids. To make ferrofluid, at least three ingredients are necessary: carrier liquid, nano-sized magnetic particles, and surfactants. Ferrofluids are primarily employed in the hard disc drive sealing process. Ferrofluids are employed as a lubricant in rotating shafts in a variety of commercial equipment. It's also utilized in speaker coils to boost the acoustic output of the speakers. In the therapy and diagnosis of cancer, ferrofluids play a critical role. A solar collector's thermal performance with displaced pipes may be measured via ferrofluids^30^. When there is an alternating magnetic field present, the viscosity of significance of magnetic fluid is crucial in optimizing the technical use of ferrofluid. The researchers looked at the viscous behavior of ferrofluid in the existence of a fixed magnetic field^31–34^. The viscosity of magnetic fluid changes as it is visible to an alternating magnetic field^35–38^. Between contracting spinning discs, the rheological properties of metal-based nanofluids have been investigated^39^. The study of magnetic viscosity is affected by the magnetic field necessitates the use of an external magnetic field. The magnetic torque and magnetization force are highly significant to examine the flow properties of fluid with magnetic properties in many forms of ferrofluid flow^40–43^. Especially in electrical engineering and electromechanics, magnetic fields are utilised in all areas of contemporary technology. Power generators and electric motors both employ rotating magnetic fields. The study of the entropy formation model's mathematical justification has been provided, along with magnetoviscous effects on ferrofluid flow in the presence of an alternating magnetic field^44^. Analysis of entropy generation and thin-film Maxwell fluid flow across a stretchable spinning disc have been studied^45,46^. The effects of nonlinear thermal radiation on a hybrid nanofluid across a cylindrical disc have been studied^47^. The binary diffusion theory was used to investigate the rotational flow of Oldroyd-B nanofluid^48^. The effect of the Hall current has been used to conduct a hybrid nanofluid over a revolving disc^49^. To improve the viscosity and thermal conductivity of the nanofluid, silver nanoparticles were utilized^50^. Considering a transverse magnetic field, nanofluid flow behavior and heat transfer across a porous shrinking surface were investigated^51^. The flow of nanofluid across a cylindrical disc in a non-axisymmetric stagnation point has been studied^52^. When a static magnetic field is present, the properties of magnetic body force and rotational viscosity in ferrofluid flow across a stretched sheet were investigated^53^. Maxwell nanofluid flow across a spinning disc with a chemical reaction was investigated^54^. The properties of heat mass transport processes have been examined using thin film deposition of nanofluid over an uneven stretched surface^55^. When there is a constant magnetic field present, the heat transfer properties of a water-based ${\text{Fe}}_{3} {\text{O}}_{4}$ nanofluid have been examined^56^.

The convective heat transfer of a magnetite-water nanofluid in the presence of an external magnetic field was experimentally examined by Azizian et al.^57^. The scientists found that as the magnetic field strength and Reynolds number rise, so do the heat transfer and pressure decrease. Goharkhan et al.^58^ carried out experimental research on the convective heat transfer of ${\text{Fe}}_{3} {\text{O}}_{4}$-water nanofluid within a heated tube in the presence of continuous and alternating magnetic fields. When the Reynolds number and nanoparticle concentration rise, an increase in heat transmission is seen. Additionally, it was discovered that when the magnetic field's intensity rises, the temperature at the walls' surface falls. Particularly, the temperature decrease is greater when an alternating magnetic field rather than a steady magnetic field is applied. The impact of a non-uniform magnetic field on convective heat transport in a Fe3O4-water ferrofluid was investigated by Sheikholeslami and Ganji^59^. Both magnetohydrodynamic and ferrohydrodynamic effects are taken into account in their analysis, and the magnetic field is created by a current-carrying wire. When the Rayleigh number, nanoparticle volume fraction, and magnetic number are increased, an increase in heat transfer is seen. Heat transfer is reduced though as the Hartmann number rises. In the presence of a non-uniform magnetic field, Gibanov et al.^60^ looked at the convective flow of water-based magnetite ferrofluid in a lid-driven cavity with a heat-conducting solid backward step. In their experiment, a magnetic wire is positioned above the top wall of the cavity and produces a non-uniform magnetic field. The intensity of convective circulation and heat transfer is found to increase as the magnetic number rises, according to the authors. The rate of heat transfer increases along with the volume fraction of nanoparticles. However, a higher Hartmann number results in a slower rate of heat transfer and fluid flow. Ghasemian et al.^61^ carried out a two-phase numerical study on the forced convective heat transfer of magnetite water-ferrofluid through a mini-channel while being affected by constant and alternating magnetic fields. The constant magnetic field is produced by current-carrying wires that are positioned below the channel, whereas the alternating magnetic field is generated by imposing rectangular wave functions on the current source of current-carrying wires, which are located above and below the channel. When the magnetic field is constant, increasing its intensity causes the flow velocity to rise over the top surface of the channel and lowers the temperature of the ferrofluid. The fluid's velocity changes along the channel's width when an alternating magnetic field is provided, which improves heat transmission. Additionally, compared to a steady magnetic field, an alternating magnetic field improves heat transmission. It was also discovered that there is a magnetic field frequency value that, as the Reynolds number rises, maximises the heat transfer improvement. Some recent work can be seen in^62–65^.

A systematic approach called lie group analysis is used to find the invariant or self-similar set of partial differential equations solutions. The technique provides a profound understanding of the physical issues that are described by partial differential equations. Lie group analysis has two applications: producing a new solution from an existing solution and discovering similar solutions for partial differential equations. The current study concentrates on the latter sort of application. This approach, which dates back to the Sophus Lie (1842–1899), is often employed to solve differential equations^66–68^. This approach was used by Jalil et al.^69^ to discover suitable similarity transformations for mixed convection flow across a stretched surface. They expanded their work to non-Newtonian fluid flow^70,71^ by utilizing Lie group analysis to identify self-similar solutions to the governing equations. Hamad et al.^72^ used Lie group analysis to explore the combined impacts of heat and mass transmission across a moving surface. Ferdows et al.^73^ investigated mixed convection over a horizontal sliding porous flat plate using the one-parameter continuous group theory approach. Ferdows et al.^74^ studied the convective effects of heat and mass transmission across a radiating stretched sheet using a specific type of Lie group of transformation (scaling transformation).

Some of the distinct attributes of nanofluid and ferrofluid have been researched in the preceding literature review. Researchers have explored the rotating flow of a nanofluid in the presence of various physical factors.

When there’s a variety of physical challenges, the rotating nanofluid flow has been investigated. In this current work, under the effects of an alternating magnetic field, hybrid nanofluid flow and heat transfer over a non-linear stretchable rotating disc are studied. The frequency of the alternating magnetic field determines how much the external magnetic field impedes the flow. We considered this problem with two different nanoparticles (${\text{Al}}_{2} {\text{O}}_{3}$–Cu) suspended in the base fluid ethylene glycol (EG). In the current physical model, the theoretical formula for rotating viscosity in the presence of an alternating magnetic field are adopted. The current model is transformed into a dimensionless form using a similarity transformation. The BVP4c is used to solve a set of nonlinear-coupled differential equations using MATLAB software. For various values of the physical parameters utilized in the problem, the findings for radial velocity, tangential velocity, axial velocity, and temperature distributions are reported.

## Mathematical formulation of rotational ferrofluid flow

**Figure 1:**
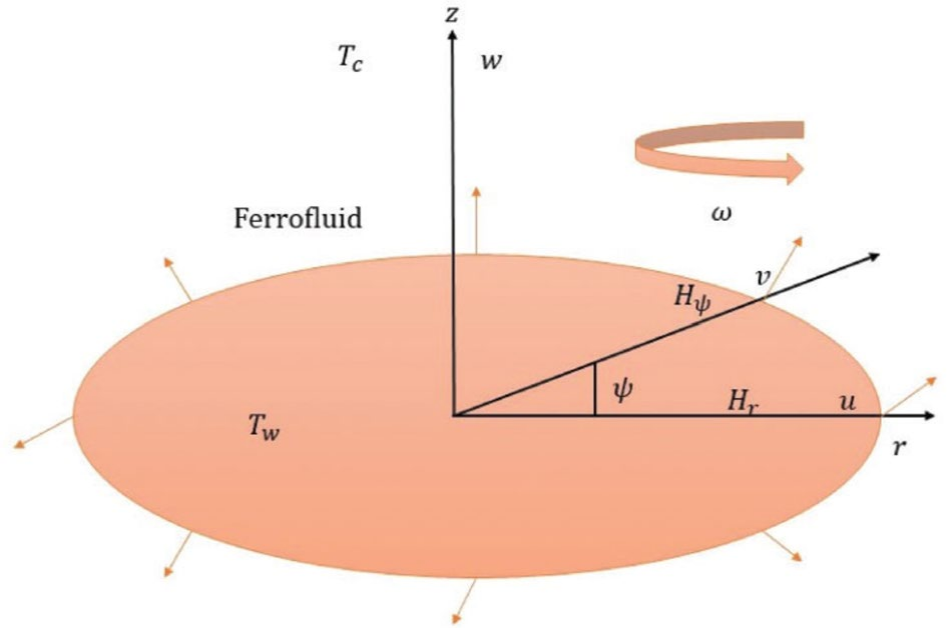
Flow across an extending disc is depicted.

Figure 1 depicts the flow arrangement. In the presence of an alternating magnetic field, the flow of a ${\text{Al}}_{2} {\text{O}}_{3} - C_{u} /EG$ ferrofluid over a radially extending disc is examined. The disc rotates at a constant angular velocity $\omega$ around the z-axis. Let the temperature on the disk’s surface is $T_{w}$ and $T_{c}$ is the temperature of Curie. The flow is deemed axisymmetric and incompressible. The constitutive equations for the motion of ferromagnetic nanofluids, magnetization equation, energy equation, and Maxwell equations are as follow^20,29^:

1 $$\rho_{n} \left( {\frac{\partial }{\partial t} + V \cdot \nabla } \right)V = - \nabla p + \mu_{n} \nabla^{2} V + \mu_{0} M\nabla H + \frac{1}{{2\tau_{s} }}\nabla \times (\omega_{p} - \Omega )$$

2 $$\nabla \cdot V = 0$$

3 $$I\frac{{d\omega_{p} }}{dt} = \mu_{0} \left( {M \times H} \right) - \frac{I}{{\tau_{s} }}\left( {\omega_{p} - \Omega } \right)$$

4 $$\frac{dM}{{dt}} = \omega_{p} \times M - \frac{1}{{\tau_{B} }}(M - M_{0} )$$

5 $$\rho_{n} c_{p} \left[ {\frac{\partial T}{{\partial t}} + (V \cdot \nabla )T} \right] = k_{n} \nabla^{2} T - \mu_{0} T\frac{\partial M}{{\partial T}} \cdot \left( {V \cdot \nabla } \right)H + \mu_{n} \phi$$

6 $$\nabla \times H = 0,\quad \nabla \cdot B = 0,$$

$(\mu_{0} = 4\pi \times 10^{ - 7} Henery/meter)$ is the magnetic permeability of the free space of the nanofluid. In comparison to a relaxation term, $\frac{{d\omega_{p} }}{dt} \ll \frac{{I\omega_{p} }}{{\tau_{s} }}$ the inertial expression is negligible. Therefore, Eq. (3) can be reduced as:

7 $$\omega_{p} = \Omega + \mu_{0} \frac{{\tau_{s} }}{I}\left( {M \times H} \right)$$

Equations (1) and (2) can be expressed as follow by using Eq. (7):

8 $$\rho_{n} \left( {\frac{\partial }{\partial t} + V \cdot \nabla } \right)V = - \nabla_{p} + \mu_{0} (M \cdot \nabla )H + \mu_{n} \nabla^{2} V + \frac{1}{2}\mu_{0} \nabla \times \left( {M \times H} \right)$$

9 $$\frac{dM}{{dt}} = \Omega \times M - \frac{1}{{\tau_{B} }}\left( {M - M_{0} } \right) - \mu_{0} \frac{{\tau_{s} }}{I}M \times \left( {M \times H} \right)$$

In the radial direction, the alternating magnetic field is applied as follows^75^:

10 $$H_{r} = H_{0} \cos \omega_{0} t\quad H_{\psi } = H_{z} = 0$$

where the applied magnetic field's angular frequency is $\omega_{0}$, $H_{0}$ signifies the magnetic field's amplitude. Consider Eq. (10) as a principle of superposition of two spinning fields: the left-hand polarized field (subscript +) and the right-hand polarized (−), then:

11 $$H_{ \pm } = \left( {H_{0} \cos \omega_{0} t, \pm H_{0} \sin \omega_{0} t,0} \right),\quad H = \frac{1}{2}\left( {H_{ + } + H_{ - } } \right)$$

Assume that the magnetization lags after the magnetic field at a certain angle $\alpha_{0}$. Then

12 $$M_{ \pm } = \left[ {M\cos \left( {\omega_{0} t - \alpha_{0} } \right), \pm M\sin \left( {\omega_{0} t - \alpha_{0} } \right),0} \right],\quad \omega_{{p_{ \pm } }} = \left( {0,0, \pm \omega_{p} } \right)$$

Using Eqs. (3), (4), (11) and (12) we get:

13 $$M = \chi^{\prime}H_{0} \cos \alpha_{0} , \omega_{p} = \left( {\mu_{0} \frac{{\tau_{s} }}{I}} \right)MH_{0} \sin \alpha_{0}, \quad \tan \alpha = \left( {\omega_{0} - \omega_{p} } \right)\tau_{B}$$

The magnetic energy to thermal energy $\left( {\xi = \frac{{mH_{0} }}{kT\sqrt 2 }} \right)$ ratio is quite tiny, and using the expression $I = 6\mu \tau_{s} \varphi$. Eliminating the angle $\alpha_{0}$ from Eq. (13):

14 $$\begin{aligned} M = & \frac{{\xi H_{0} }}{{\sqrt {1 + \omega_{0}^{2} \tau_{B}^{2} } }},\; \omega_{p} = & \omega_{0} \frac{{\xi^{2} /3}}{{1 + \omega_{0}^{2} \tau_{B}^{2} }},\; \tan \alpha_{0} = & \omega_{0} \tau_{B} \left( {1 - \frac{{\xi^{2} /3}}{{1 + \omega_{0}^{2} \tau_{B}^{2} }}} \right) \\ \end{aligned}$$

$\frac{{H_{0} }}{\sqrt 2 }$ is the root mean square value of the $H_{0} \cos \omega_{0} t$ and $\alpha_{0}$ is the magnetic field's phase angle with the magnetization. Taking into account the hydrodynamical vortex $\Omega = \left( {0,0,\Omega } \right)$ and the spinning magnetic field, magnetization's tangential components are as follow^75^:

15 $$M_{\psi }^{ + } = \chi^{\prime}H_{0} \cos \alpha_{0 + } \sin \left( {\omega_{0} t - \alpha_{0 + } } \right) = \frac{{\chi^{\prime}H_{0} }}{{1 + (\omega_{0} - \Omega )^{2} \tau_{B}^{2} }}\left[ {\sin \omega_{0} t - \left( {\omega_{0} - \Omega } \right)\tau_{B} \cos \omega_{0} t} \right]$$

16 $$M_{\psi }^{ - } = - \chi^{\prime}H_{0} \cos \alpha_{0 - } \sin \left( {\omega_{0} t - \alpha_{0 - } } \right) = - \frac{{\chi^{\prime}H_{0} }}{{1 + (\omega_{0} + \Omega )^{2} \tau_{B}^{2} }}\left[ {\sin \omega_{0} t - \left( {\omega_{0} + \Omega } \right)\tau_{B} \cos \omega_{0} t} \right]$$

The tangential component of magnetization is: if the field becomes linearly polarized along the radial direction

17 $$M_{\psi } = \frac{1}{2}\left( {M_{\psi }^{ + } + M_{\psi }^{ - } } \right) = \Omega \tau_{B} H_{0} \cos^{2} \alpha \cos \left( {\omega_{0} t - 2\alpha_{0} } \right)$$

Along the z-axis, the magnetic torque operates on the fluid as follows^75^:

18 $$\begin{aligned} \mu_{0} \left( {M \times H} \right) = & - \mu_{0} \Omega \tau_{B} \frac{\chi }{{\mu_{0} }}H_{0}^{2} \cos^{2} \alpha_{0} \cos \omega_{0} t\cos \left( {\omega_{0} t - 2\alpha_{0} } \right) \\ = & - 2\Omega \mu_{n} \varphi \xi^{2} \cos^{2} \alpha_{0} \times \left( {\cos^{2} \omega_{0} t\cos 2\alpha_{0} + \sin \omega_{0} t\cos \omega_{0} t\sin 2\alpha_{0} } \right) \\ \end{aligned}$$

Taking the average of Eq. (19) throughout the field fluctuation period $\frac{2\pi }{{\omega_{0} }}$,

19 $$\mu_{0} \left( {\overline{M \times H} } \right) = - \Omega \mu_{n} \varphi \xi^{2} \cos^{2} \alpha_{0} \cos 2\alpha_{0}$$

20 $$\begin{aligned} \frac{{\mu_{0} }}{2}\nabla \times \left( {\overline{M \times H} } \right) = & \frac{1}{2}\nabla \times - \Omega \mu_{n} \varphi \xi^{2} \cos^{2} \alpha_{0} \cos 2\alpha_{0} \\ = & \frac{1}{4}\mu_{n} \varphi \xi^{2} \cos^{2} \alpha_{0} \cos 2\alpha_{0} \nabla^{2} V \\ = & \frac{1}{4}\mu_{n} \varphi \xi^{2} \left( {\frac{{1 - \omega_{0}^{2} \tau_{B}^{2} }}{{\left( {1 + \omega_{0}^{2} \tau_{B}^{2} } \right)^{2} }}} \right)\nabla^{2} V \\ \end{aligned}$$

Because of the oscillating magnetic field, the expression $\frac{1}{4}\varphi \xi^{2} \left( {\frac{{1 - \omega_{0}^{2} \tau_{B}^{2} }}{{\left( {1 + \omega_{0}^{2} \tau_{B}^{2} } \right)^{2} }}} \right)$ is termed rotating viscosity. It is determined by the magnetic field's strength $\xi$ as well as the magnetic field's frequency. For $\omega_{0} \tau_{B} > 1$, the rotating viscosity decreases. This is referred to as a negative viscosity impact. If $\omega_{0} \tau_{B} = 1$, the rotating viscosity does not influence the fluid. If $\omega_{0} \tau_{B} < 1$, the fluid is subjected to increased resistance due to the oscillating magnetic field. In the limiting case $\omega_{0} \tau_{B} \to \infty$, the impact of rotating viscosity vanishes due to the nanoparticles in the fluid no longer sensing the magnetic field.

The applied magnetic field has a scalar potential^75^

21 $$\gamma = \frac{{\xi_{0} \cos \omega_{0} t}}{2\pi }\frac{\cos \psi }{r}$$

The magnetic field components in radial and tangential directions can be written as^75^

22 $$H_{r} = - \frac{\partial \gamma }{{\partial r}} = \frac{{\xi_{0} \cos \omega_{0} t}}{2\pi }\frac{\cos \psi }{{r^{2} }},\quad H_{\psi } = - \frac{\partial \gamma }{{\partial \theta }} = \frac{{\xi_{0} \cos \omega_{0} t}}{2\pi }\frac{r\sin \psi }{{r^{2} }}$$

The intensity of the entire magnetic field can be calculated as follows^75^

23 $$H = \sqrt {\left( {H_{r} } \right)^{2} + \left( {\frac{1}{r}H_{\psi } } \right)^{2} } = \frac{{\xi_{0} \cos \omega_{0} t}}{2\pi }\frac{1}{{r^{2} }}$$

In the radial and tangential directions, the following are the rates of change in magnetic field intensity^75^

24 $$\frac{\partial H}{{\partial r}} = \frac{{ - \xi_{0} \cos \omega_{0} t}}{{\pi r^{3} }},\quad \frac{\partial H}{{\partial \psi }} = 0$$

The radial and tangential magnetization forces can be represented as follows^75^

25 $$\mu_{0} M\frac{\partial H}{{\partial r}} = - \mu_{0} M\frac{{\xi_{0} \cos \omega_{0} t}}{{\pi r^{3} }},\quad \mu_{0} \frac{M}{r}\frac{\partial H}{{\partial \psi }} = 0$$

The temperature has a linear effect on magnetization, as follows^75^

26 $$M = K^{a} \left( {T_{c} - T} \right)$$

In the above equation, the pyro magnetic coefficient is denoted by $K^{a}$ and the Curie temperature is denoted by $T_{c}$. Equations (2), (5), and (8) can be written in cylindrical form using Eqs. (20, 25), and (26):

27 $$\frac{{\partial \tilde{u}}}{{\partial \tilde{r}}} + \frac{{\tilde{u}}}{{\tilde{r}}} + \frac{{\partial \tilde{w}}}{{\partial \tilde{z}}} = 0$$

28 $$\begin{aligned} \rho_{hnf} \left[ {\tilde{u}\frac{{\partial \tilde{u}}}{{\partial \tilde{r}}} + \tilde{w}\frac{{\partial \tilde{u}}}{{\partial \tilde{z}}} - \frac{{\tilde{v}^{2} }}{{\tilde{r}}}} \right] = & - \frac{{\partial \tilde{p}}}{{\partial \tilde{r}}} + \mu_{hnf} \left( {1 + \frac{1}{4}\varphi \xi^{2} \left( {\frac{{1 - \omega_{0}^{2} \tau_{B}^{2} }}{{\left( {1 + \omega_{0}^{2} \tau_{B}^{2} } \right)^{2} }}} \right)} \right) \\ & \quad \times \left\{ {\frac{{\partial^{2} \tilde{u}}}{{\partial \tilde{z}^{2} }} + \frac{1}{{\tilde{r}}}\frac{{\partial \tilde{u}}}{{\partial \tilde{r}}} - \frac{{\tilde{u}}}{{\tilde{r}^{2} }} + \frac{{\partial^{2} \tilde{u}}}{{\partial \tilde{r}^{2} }}} \right\} - \mu_{0} K^{a} \left( {T_{c} - T} \right)\frac{{\xi_{0} \cos \omega_{0} t}}{\pi }\frac{1}{{\tilde{r}^{3} }} \\ \end{aligned}$$

29 $$\rho_{hnf} \left[ {\tilde{u}\frac{{\partial \tilde{v}}}{{\partial \tilde{r}}} + \tilde{w}\frac{{\partial \tilde{v}}}{{\partial \tilde{z}}} + \frac{{\tilde{u}\tilde{v}}}{{\tilde{r}}}} \right] = \mu_{hnf} \left( {1 + \frac{1}{4}\varphi \xi^{2} \left( {\frac{{1 - \omega_{0}^{2} \tau_{B}^{2} }}{{\left( {1 + \omega_{0}^{2} \tau_{B}^{2} } \right)^{2} }}} \right)} \right) \times \left\{ {\frac{{\partial^{2} \tilde{v}}}{{\partial \tilde{z}^{2} }} + \frac{1}{{\tilde{r}}}\frac{{\partial \tilde{v}}}{{\partial \tilde{r}}} - \frac{{\tilde{v}}}{{\tilde{r}^{2} }} + \frac{{\partial^{2} \tilde{v}}}{{\partial \tilde{r}^{2} }}} \right\}$$

30 $$\tilde{u}\frac{{\partial \tilde{w}}}{{\partial \tilde{r}}} + \tilde{w}\frac{{\partial \tilde{w}}}{{\partial \tilde{z}}} = - \frac{1}{\rho }\frac{{\partial \tilde{p}}}{{\partial \tilde{z}}} + \upsilon \left\{ {\frac{{\partial^{2} \tilde{w}}}{{\partial \tilde{r}^{2} }} + \frac{1}{{\tilde{r}}}\frac{{\partial \tilde{w}}}{{\partial \tilde{r}}} + \frac{{\partial^{2} \tilde{w}}}{{\partial \tilde{z}^{2} }}} \right\} \times \left( {1 + \frac{1}{4}\varphi \xi^{2} \left( {\frac{{1 - \omega_{0}^{2} \tau_{B}^{2} }}{{\left( {1 + \omega_{0}^{2} \tau_{B}^{2} } \right)^{2} }}} \right)} \right)$$

31 $$\begin{aligned} & \rho_{hnf} c_{p} \left( {\tilde{u}\frac{{\partial \tilde{T}}}{{\partial \tilde{r}}} + \tilde{w}\frac{{\partial \tilde{T}}}{{\partial \tilde{z}}}} \right) + \mu_{0} \tilde{T}\frac{{\partial \tilde{M}}}{{\partial \tilde{T}}}\left( {\tilde{u}\frac{{\partial \tilde{H}}}{{\partial \tilde{r}}} + \frac{1}{{\tilde{r}}}\tilde{v}\frac{{\partial \tilde{H}}}{{\partial \tilde{\psi }}}} \right) \\ & \quad = k_{hnf} \left\{ {\frac{1}{{\tilde{r}}}\frac{\partial }{{\partial \tilde{r}}}\left( {\tilde{r}\frac{{\partial \tilde{T}}}{{\partial \tilde{r}}}} \right) + \frac{{\partial^{2} \tilde{T}}}{{\partial \tilde{z}^{2} }}} \right\} + \mu_{hnf} \left\{ {\left( {\frac{{\partial \tilde{u}}}{{\partial \tilde{z}}}} \right)^{2} + \left( {\frac{{\partial \tilde{v}}}{{\partial \tilde{z}}}} \right)^{2} } \right\} \\ \end{aligned}$$

32 $$\begin{gathered} \tilde{z} = 0,\quad \tilde{u} = \alpha \Omega \tilde{r}u_{w} \left( {{\raise0.7ex\hbox{${\tilde{r}}$} \!\mathord{\left/ {\vphantom {{\tilde{r}} R}}\right.\kern-\nulldelimiterspace} \!\lower0.7ex\hbox{$R$}}} \right),\quad \tilde{v} = \Omega \tilde{r}v_{w} \left( {{{\tilde{r}} \mathord{\left/ {\vphantom {{\tilde{r}} R}} \right. \kern-\nulldelimiterspace} R}} \right),\quad \tilde{w} = 0, \hfill \\ \tilde{T} = \tilde{T}_{w} ,\quad \tilde{z} \to \infty ,\quad \tilde{u} = 0,\quad \tilde{v} = 0,\quad \tilde{T} = \tilde{T}_{\infty } . \hfill \\ \end{gathered}$$

## Boundary layer equations

Incorporating non-dimensional variables into the governing Eqs. (27)–(32) is a practical way to find boundary layer equations. For the current problem, we examine the following non-dimensional variables:

33 $$\begin{gathered} r = \frac{{\tilde{r}}}{R},\quad z = \frac{{\tilde{z}}}{R}{\text{Re}}^{{{1 \mathord{\left/ {\vphantom {1 2}} \right. \kern-\nulldelimiterspace} 2}}} ,\quad u = \frac{{\tilde{u}}}{\Omega R},\quad v = \frac{{\tilde{v}}}{\Omega R}, \hfill \\ w = \frac{{\tilde{w}}}{\Omega R}{\text{Re}}^{{{1 \mathord{\left/ {\vphantom {1 2}} \right. \kern-\nulldelimiterspace} 2}}} ,\quad p = \frac{{\tilde{p}}}{{\rho \left( {\Omega R} \right)^{2} }},\quad T = \frac{{\tilde{T} - \tilde{T}_{\infty } }}{{T_{w} - T_{\infty } }} \hfill \\ \end{gathered}$$

where ${\text{Re}} = \frac{{\Omega R^{2} }}{\upsilon }$ is Reynolds number, the reference length is designated by $R$ and the reference temperature is $\left( {T_{w} - T_{\infty } } \right)$. It's worth noting that in the axial direction, the analogous scales are smaller by a factor ${\text{Re}}^{{{{ - 1} \mathord{\left/ {\vphantom {{ - 1} 2}} \right. \kern-\nulldelimiterspace} 2}}} ,$ As a result, ${\text{Re}} \gg 1$ is implicitly foreshadowed. The controlling Eqs. (27)–(32) are now transformed into a dimensionless version, as follows:

34 $$\frac{\partial u}{{\partial r}} + \frac{u}{r} + \frac{\partial w}{{\partial z}} = 0$$

35 $$\begin{aligned} u\frac{\partial u}{{\partial r}} + w\frac{\partial u}{{\partial z}} - \frac{{v^{2} }}{r} = & \frac{{e_{2} }}{{e_{1} }}\frac{1}{{\text{Re}}}\left( {1 + \frac{1}{4}\varphi \xi^{2} \left( {\frac{{1 - \omega_{0}^{2} \tau_{B}^{2} }}{{\left( {1 + \omega_{0}^{2} \tau_{B}^{2} } \right)^{2} }}} \right)} \right) \\ & \quad \times \left( {{\text{Re}} \frac{{\partial^{2} u}}{{\partial z^{2} }} + \frac{1}{r}\frac{\partial u}{{\partial r}} - \frac{u}{{r^{2} }} + \frac{{\partial^{2} u}}{{\partial r^{2} }}} \right) - \frac{{\mu_{0} k^{a} \left( {T_{c} - T} \right)\xi_{0} \cos \omega_{0} t}}{{e_{1} \cdot \rho_{f} \pi r^{3} R^{4} \Omega^{2} }} \\ \end{aligned}$$

36 $$u\frac{\partial v}{{\partial r}} + w\frac{\partial v}{{\partial z}} - \frac{uv}{r} = \frac{{e_{2} }}{{e_{1} }}\frac{1}{{\text{Re}}}\left( {1 + \frac{1}{4}\varphi \xi^{2} \left( {\frac{{1 - \omega_{0}^{2} \tau_{B}^{2} }}{{\left( {1 + \omega_{0}^{2} \tau_{B}^{2} } \right)^{2} }}} \right)} \right) \times \left( {{\text{Re}} \frac{{\partial^{2} v}}{{\partial z^{2} }} + \frac{1}{r}\frac{\partial v}{{\partial r}} + \frac{{\partial^{2} v}}{{\partial r^{2} }} - \frac{v}{{r^{2} }}} \right)$$

37 $$\frac{1}{{\text{Re}}}\left( {u\frac{\partial w}{{\partial r}} + w\frac{\partial w}{{\partial z}}} \right) = - \frac{\partial p}{{\partial z}} + \frac{1}{{{\text{Re}}^{2} }}\left\{ {\frac{{\partial^{2} w}}{{\partial r^{2} }} + \frac{1}{r}\frac{\partial w}{{\partial r}} + {\text{Re}} \frac{{\partial^{2} w}}{{\partial z^{2} }}} \right\} \times \left( {1 + \frac{1}{4}\varphi \xi^{2} \left( {\frac{{1 - \omega_{0}^{2} \tau_{B}^{2} }}{{\left( {1 + \omega_{0}^{2} \tau_{B}^{2} } \right)^{2} }}} \right)} \right)$$

38 $$\begin{aligned} & u\frac{\partial T}{{\partial r}} + w\frac{\partial T}{{\partial z}} + \frac{{\mu_{0} k^{a} Ru\xi_{0} \cos \omega_{0} t}}{{e_{1} \rho_{f} c_{p} \pi r^{3} \left( {T_{w} - T_{\infty } } \right)}}\left[ {T\left( {T_{w} - T_{\infty } } \right) + T_{\infty } } \right] \\ & \quad = \frac{{e_{3} }}{{e_{1} }}\frac{1}{{\Pr \cdot {\text{Re}} }}\left\{ {\frac{1}{r}\frac{\partial T}{{\partial r}} + \frac{{\partial^{2} T}}{{\partial r^{2} }} + {\text{Re}} \frac{{\partial^{2} T}}{{\partial z^{2} }}} \right\} + \frac{{e_{2} }}{{e_{1} }}\frac{{\upsilon_{f} {\text{Re}} }}{{c_{p} \left( {T_{w} - T_{\infty } } \right)}}\left\{ {\left( {\frac{\partial u}{{\partial z}}} \right)^{2} + \left( {\frac{\partial v}{{\partial z}}} \right)^{2} } \right\} \\ \end{aligned}$$

where Prandtl number is $\Pr = {\upsilon \mathord{\left/ {\vphantom {\upsilon {\alpha_{T} }}} \right. \kern-\nulldelimiterspace} {\alpha_{T} }}$.

When the Reynolds number is high, i.e., ${\text{Re}} \to \infty$, In dimensionless form, the resultant boundary layer equations are as follows:

39 $$\frac{\partial u}{{\partial r}} + \frac{u}{r} + \frac{\partial w}{{\partial z}} = 0$$

40 $$u\frac{\partial u}{{\partial r}} + w\frac{\partial u}{{\partial z}} - \frac{{v^{2} }}{r} = \frac{{e_{2} }}{{e_{1} }}\left( {1 + \frac{1}{4}\varphi \xi^{2} \left( {\frac{{1 - \omega_{0}^{2} \tau_{B}^{2} }}{{\left( {1 + \omega_{0}^{2} \tau_{B}^{2} } \right)^{2} }}} \right)} \right) \times \left( {\frac{{\partial^{2} u}}{{\partial z^{2} }}} \right) - \frac{{\mu_{0} k^{a} \left( {T_{c} - T} \right)\xi_{0} \cos \omega_{0} t}}{{e_{1} \cdot \rho_{f} \pi r^{3} R^{4} \Omega^{2} }}$$

41 $$u\frac{\partial v}{{\partial r}} + w\frac{\partial v}{{\partial z}} - \frac{uv}{r} = \frac{{e_{2} }}{{e_{1} }}\left( {1 + \frac{1}{4}\varphi \xi^{2} \left( {\frac{{1 - \omega_{0}^{2} \tau_{B}^{2} }}{{\left( {1 + \omega_{0}^{2} \tau_{B}^{2} } \right)^{2} }}} \right)} \right) \times \left( {\frac{{\partial^{2} v}}{{\partial z^{2} }}} \right)$$

42 $$- \frac{\partial p}{{\partial z}} = 0$$

43 $$u\frac{\partial T}{{\partial r}} + w\frac{\partial T}{{\partial z}} + \frac{{\mu_{0} k^{a} Ru\xi_{0} \cos \omega_{0} t}}{{e_{1} \rho_{f} c_{p} \pi r^{3} \left( {T_{w} - T_{\infty } } \right)}}\left[ {T\left( {T_{w} - T_{\infty } } \right) + T_{\infty } } \right] = \frac{{e_{3} }}{{e_{1} }}\frac{1}{\Pr }\left\{ {\frac{{\partial^{2} T}}{{\partial z^{2} }}} \right\}$$

The following are the ferrofluid flow boundary conditions across a stretched disc:

44 $$\begin{gathered} z = 0;\quad u = ar^{m} ,\quad v = ar^{m} ,\quad w = 0,\quad T = T_{w} , \hfill \\ z \to \infty ,\quad u \to 0,\quad v \to 0,\quad T \to T_{c} \hfill \\ \end{gathered}$$

Von Karman^1^ suggested the similarity transformation, which has the characteristic that pressure depends only on z. According to Eq. (42), the pressure in the axial direction is constant in the boundary layer area. The logical conclusion from this is that the pressure term within the boundary layer is simply constant and, as a result, identical to the ambient pressure.

Density $\left( {\rho_{nf} } \right)$, viscosity $\left( {\mu_{nf} } \right)$ and thermal diffusivity $\left( {\alpha_{nf} } \right)$ of nanofluid are^75^,

45 $$\begin{gathered} \rho_{nf} = \left( {1 - \varphi } \right)\rho_{f} + \varphi \rho_{s} ,\quad \mu_{nf} = \frac{{\mu_{f} }}{{\left( {1 - \varphi } \right)^{2.5} }},\quad \alpha_{nf} = \frac{{k_{nf} }}{{\left( {\rho c_{p} } \right)_{nf} }}, \hfill \\ \left( {\rho c_{p} } \right)_{nf} = \left( {1 - \varphi } \right)\left( {\rho c_{p} } \right)_{f} + \varphi \left( {\rho c_{p} } \right)_{s} ,\quad \frac{{k_{nf} }}{{k_{f} }} = \frac{{k_{s} + 2k_{f} - 2\varphi \left( {k_{f} - k_{s} } \right)}}{{k_{s} + 2k_{f} + \varphi \left( {k_{f} - k_{s} } \right)}} \hfill \\ \end{gathered}$$

The thermophysical properties $\rho_{hnf}$, $\left( {\rho c_{p} } \right)_{hnf}$, $\mu_{hnf}$ and $k_{hnf}$ are defined for hybrid nanofluid (Al_2_O_3_-Cu/EG) are defined as^76^,

$$\rho_{hnf} = \left( {1 - \phi_{2} } \right)\left[ {\left( {1 - \phi_{1} } \right)\rho_{f} + \phi_{1} \rho_{s1} } \right] + \phi_{2} \rho_{s2} ,$$

$$(\rho c_{p} )_{hnf} = \left( {1 - \phi_{2} } \right)\left[ {\left( {1 - \phi_{1} } \right)(\rho c_{p} )_{f} + \phi_{1} (\rho c_{p} )_{s1} } \right] + \phi_{2} (\rho c_{p} )_{s2} ,$$

$$\frac{{\mu_{hnf} }}{{\mu_{f} }} = \frac{1}{{\left( {1 - \varphi_{1} } \right)^{2.5} \left( {1 - \varphi_{2} } \right)^{2.5} }},\frac{{k_{hnf} }}{{k_{bf} }} = \frac{{k_{s2} + 2k_{bf} - 2\varphi_{2} \left( {k_{bf} - k_{s2} } \right)}}{{k_{s2} + 2k_{bf} + \varphi_{2} (k_{bf} - k_{s2} )}}$$

where

46 $$\frac{{k_{bf} }}{{k_{f} }} = \frac{{k_{s1} + 2k_{f} - 2\varphi_{1} \left( {k_{f} - k_{s1} } \right)}}{{k_{s1} + 2k_{f} + \varphi_{1} \left( {k_{f} - k_{s1} } \right)}}$$

Using base fluid, Table 1 shows the physical characteristics of the carrier liquid and nanoparticles.

**Table 1:** Thermophysical characteristics of nanoparticles and base fluid^76^.

Physical properties	Base fluid (EG)	Alumina (${\text{Al}}_{2} {\text{O}}_{3}$)	Cu
${{c_{p} } \mathord{\left/ {\vphantom {{c_{p} } {\left( {\frac{{\text{J}}}{{{\text{kg}}.{\text{k}}}}} \right)}}} \right. \kern-\nulldelimiterspace} {\left( {\frac{{\text{J}}}{{{\text{kg}}.{\text{k}}}}} \right)}}$	2415	765	385
${\rho \mathord{\left/ {\vphantom {\rho {\left( {\frac{{{\text{kg}}}}{{{\text{m}}^{3} }}} \right)}}} \right. \kern-\nulldelimiterspace} {\left( {\frac{{{\text{kg}}}}{{{\text{m}}^{3} }}} \right)}}$	1114.0	3970	8933
${k \mathord{\left/ {\vphantom {k {\left( {\frac{{\text{W}}}{{{\text{m}}.{\text{k}}}}} \right)}}} \right. \kern-\nulldelimiterspace} {\left( {\frac{{\text{W}}}{{{\text{m}}.{\text{k}}}}} \right)}}$	0.2520	40	400
${\sigma \mathord{\left/ {\vphantom {\sigma {\left( {\frac{{\text{s}}}{{\text{m}}}} \right)}}} \right. \kern-\nulldelimiterspace} {\left( {\frac{{\text{s}}}{{\text{m}}}} \right)}}$	$5.50 \times 10^{ - 6}$	$59.6 \times 10^{6}$	$35 \times 10^{6}$

## Similarity transformations

Using the following similarity transformation,

47 $$\begin{aligned} & \eta = \frac{z}{{r^{{{\raise0.7ex\hbox{${1 - m}$} \!\mathord{\left/ {\vphantom {{1 - m} 2}}\right.\kern-\nulldelimiterspace} \!\lower0.7ex\hbox{$2$}}}} }},\;u = r^{m} f^{\prime}\left( \eta \right),\;v = r^{m} G\left( \eta \right), \\ & w = - r^{{{\raise0.7ex\hbox{${m - 1}$} \!\mathord{\left/ {\vphantom {{m - 1} 2}}\right.\kern-\nulldelimiterspace} \!\lower0.7ex\hbox{$2$}}}} \left( {\frac{{m + 3}}{2}} \right)f\left( \eta \right) - r^{{{\raise0.7ex\hbox{${m - 1}$} \!\mathord{\left/ {\vphantom {{m - 1} 2}}\right.\kern-\nulldelimiterspace} \!\lower0.7ex\hbox{$2$}}}} \left( {\frac{{m - 1}}{2}} \right)\eta f^{\prime}\left( \eta \right),\;\theta \left( \eta \right) = \frac{{T - T_{\infty } }}{{T_{w} - T_{\infty } }}. \\ \end{aligned}$$

The continuity Eq. (39) is immediately met by using the similarity transformation (47), and the boundary layer issue (40)–(43) is readily translated into a self-similar form:

48 $$\frac{{e_{2} }}{{e_{1} }}f^{\prime\prime\prime}\left( \eta \right)\left( {1 + \frac{1}{4}\varphi \xi^{2} \left( {\frac{{1 - \omega_{0}^{2} \tau_{B}^{2} }}{{\left( {1 + \omega_{0}^{2} \tau_{B}^{2} } \right)^{2} }}} \right)} \right) - mf^{{\prime}{2}} \left( \eta \right) + \left( {\frac{m + 3}{2}} \right)f\left( \eta \right)f^{\prime\prime}\left( \eta \right) - \frac{1}{{e_{1} }}\beta = 0$$

49 $$\frac{{e_{2} }}{{e_{1} }}G^{\prime\prime}\left( \eta \right)\left( {1 + \frac{1}{4}\varphi \xi^{2} \left( {\frac{{1 - \omega_{0}^{2} \tau_{B}^{2} }}{{\left( {1 + \omega_{0}^{2} \tau_{B}^{2} } \right)^{2} }}} \right)} \right) - (m + 1)G\left( \eta \right)f^{\prime}\left( \eta \right) + \left( {\frac{m + 3}{2}} \right)f\left( \eta \right)G^{\prime}\left( \eta \right) = 0$$

50 $$\frac{{e_{3} }}{{e_{1} }}\frac{1}{\Pr }\theta^{\prime\prime}\left( \eta \right) + \left( {\frac{m + 3}{2}} \right)f\left( \eta \right)\theta^{\prime}\left( \eta \right) - \frac{{\beta_{2} }}{{e_{1} }}Rf^{\prime}\left( \eta \right)\theta \left( \eta \right) - \frac{{\beta_{1} }}{{e_{1} }}Rf^{\prime}\left( \eta \right) - \frac{{\beta_{3} }}{{e_{1} }}Rf^{\prime}\left( \eta \right) = 0$$

The following are the boundary conditions:

51 $$\begin{aligned} & f^{\prime}\left( 0 \right) = 1,\quad G\left( 0 \right) = 1,\quad f\left( 0 \right) = 0,\quad \theta \left( 0 \right) = 1,\quad {\text{at}}\quad \eta = 0 \\ & f^{\prime}\left( \infty \right) = 0,\quad G\left( \infty \right) = 0,\quad \theta \left( \infty \right) = 0,\quad {\text{at}}\quad \eta \to \infty . \\ \end{aligned}$$

The dimensionless quantities are used as follows:

52 $$\begin{aligned} & \beta = \frac{{\mu _{0} k^{a} \left( {T_{\infty } - T} \right)\xi _{0} \cos \omega _{0} t}}{{\rho _{f} \pi r^{3} R^{4} \Omega ^{2} }},\;\beta _{1} = \frac{{\mu _{0} k^{a} T_{\infty } \xi _{0} \cos \omega _{0} t}}{{e_{1} \rho _{f} c_{p} \pi r^{2} \left( {T_{w} - T_{\infty } } \right)}},\;\beta _{2} = \frac{{\mu _{0} k^{a} \xi _{0} \cos \omega _{0} t}}{{e_{1} \rho _{f} c_{p} \pi r^{2} }}, \\ & \beta _{3} = \frac{{\mu _{0} K^{a} R\xi _{0} T_{\infty } \cos \omega _{0} t}}{{e_{1} \cdot \rho _{f} c_{p} \pi r^{2} (T_{w} - T_{\infty } )^{2} }},\;\Pr = \frac{\nu }{{\alpha _{f} }},\;\alpha _{f} = \frac{k}{{\rho c_{p} }} \\ \end{aligned}$$

the ferromagnetic interaction numbers are $\beta ,\beta_{1} ,\beta_{2}$ and $\beta_{3}$, Prandtl number is denoted by Pr, the thermal diffusivity is $\alpha_{f} = \frac{k}{{\rho c_{p} }}$ in Eq. (52). The parameter $\alpha$ is the disc stretching parameter, which is a constant. The shear stress on the disk’s surface $\left( {\tau_{s} } \right)$, wall $\left( {\tau_{w} } \right)$ and flow of heat from the walls can be computed as follows:

53 $$\tau_{s} = \mu_{n} \left[ {\frac{\partial w}{{\partial r}} + \frac{\partial u}{{\partial z}}} \right]_{z = 0} ,\tau_{w} = \mu_{n} \left[ {\frac{1}{r}\frac{\partial w}{{\partial \theta }} + \frac{\partial v}{{\partial z}}} \right]_{z = 0}$$

## Results and discussion

**Figure 2:**
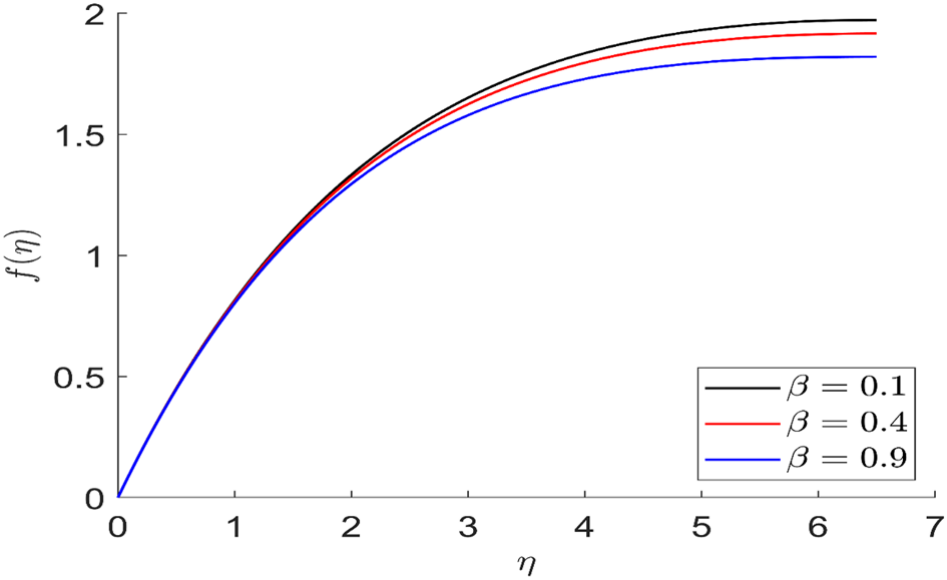
Representation of axial velocity $f\left( \eta \right)$ for the different values of $\beta$ with R = 3, n = 3, v = 0.3, $\phi$ = 0.3, Pr = 0.6, $\phi_{1}$ = 0.1, $\phi_{2}$ = 0.8, m = 1.5, $\beta_{1}$ = 0.2, $\beta_{2}$ = 0.5, $\beta_{3}$ = 0.5, u = 0.2, $\xi$ = 0.2.

**Figure 3:**
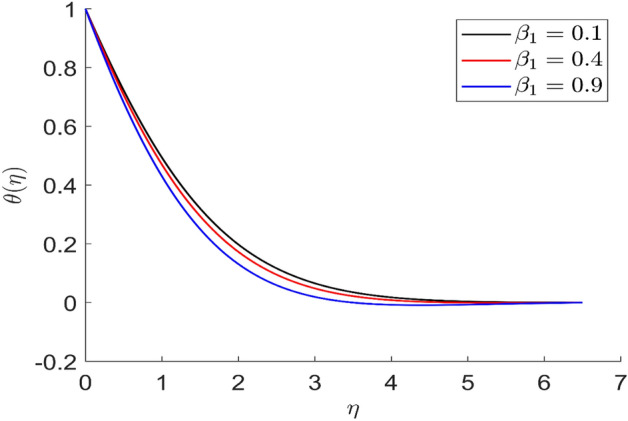
Representation of temperature $\theta \left( \eta \right)$ for the different values of $\beta_{1} \left( \eta \right)$ with R = 3, n = 3, v = 0.3, $\phi$ = 0.3, Pr = 0.6, $\phi_{1}$ = 0.1, $\phi_{2}$ = 0.8, m = 1.5, $\beta$ = 0.4, $\beta_{2}$ = 0.5, $\beta_{3}$ = 0.5, u = 0.2, $\xi$ = 0.2.

**Figure 4:**
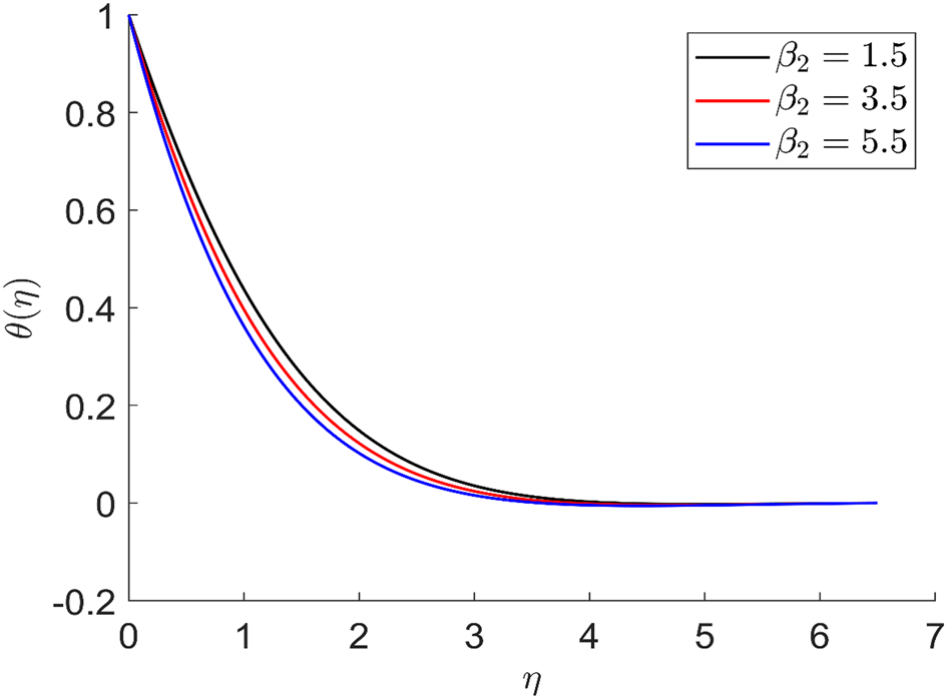
Representation of temperature $\theta \left( \eta \right)$ for the different values of $\beta_{2} \left( \eta \right)$ with R = 3, n = 3, v = 0.7, $\phi$ = 0.3, Pr = 0.6, $\phi_{1}$ = 0.1, $\phi_{2}$ = 0.8, m = 1.5, $\beta$ = 0.4, $\beta_{1}$ = 0.5, $\beta_{3}$ = 0.5, u = 0.2, $\xi$ = 0.2.

**Figure 5:**
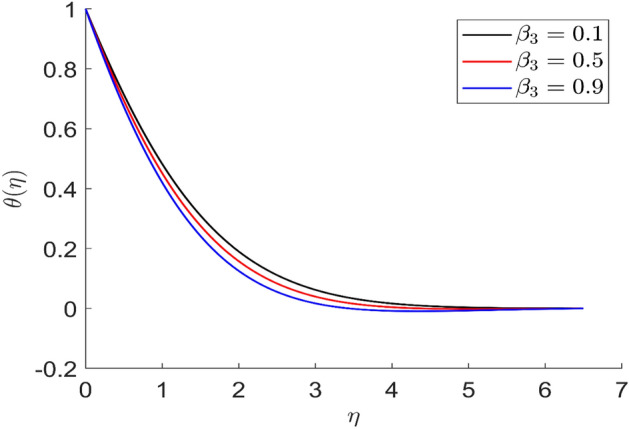
Representation of temperature $\theta \left( \eta \right)$ for the different values of $\beta_{3} \left( \eta \right)$ with R = 3, n = 3, v = 0.7, $\phi$ = 0.3, Pr = 0.6, $\phi_{1}$ = 0.1, $\phi_{2}$ = 0.8, m = 1.5, $\beta$ = 0.4, $\beta_{1}$ = 0.5, $\beta_{2}$ = 0.9, u = 0.2, $\xi$ = 0.2.

**Figure 6:**
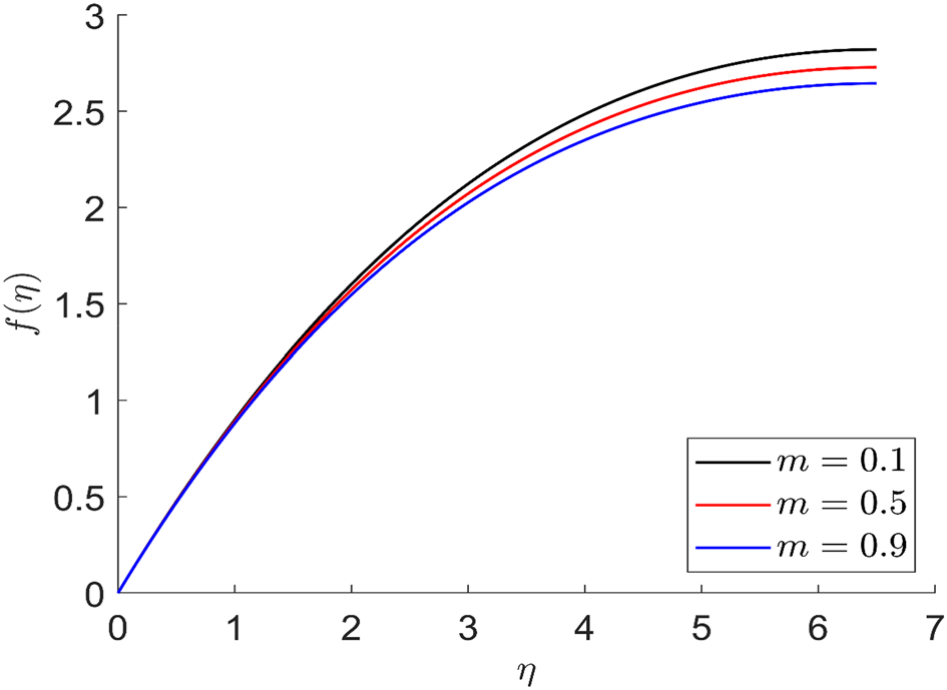
Representation of axial velocity $f\left( \eta \right)$ for the different values of $m$ with R = 3, n = 3, v = 0.1, $\phi$ = 0.2, Pr = 0.6, $\phi_{1}$ = 0.4, $\phi_{2}$ = 0.8, $\beta_{3}$ = 0.5, $\beta$ = 0.4, $\beta_{1}$ = 0.5, $\beta_{2}$ = 0.9, u = 0.2, $\xi$ = 0.2.

**Figure 7:**
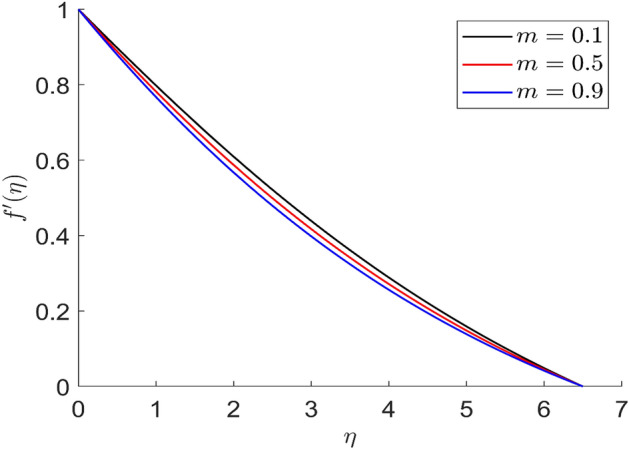
Representation of radial velocity $f^{\prime}\left( \eta \right)$ for the different values of $m$ with R = 3, n = 3, v = 0.1, $\phi$ = 0.2, Pr = 0.6, $\phi_{1}$ = 0.4, $\phi_{2}$ = 0.8, $\beta_{3}$ = 0.5, $\beta$ = 0.4, $\beta_{1}$ = 0.5, $\beta_{2}$ = 0.9, u = 0.2, $\xi$ = 0.2.

**Figure 8:**
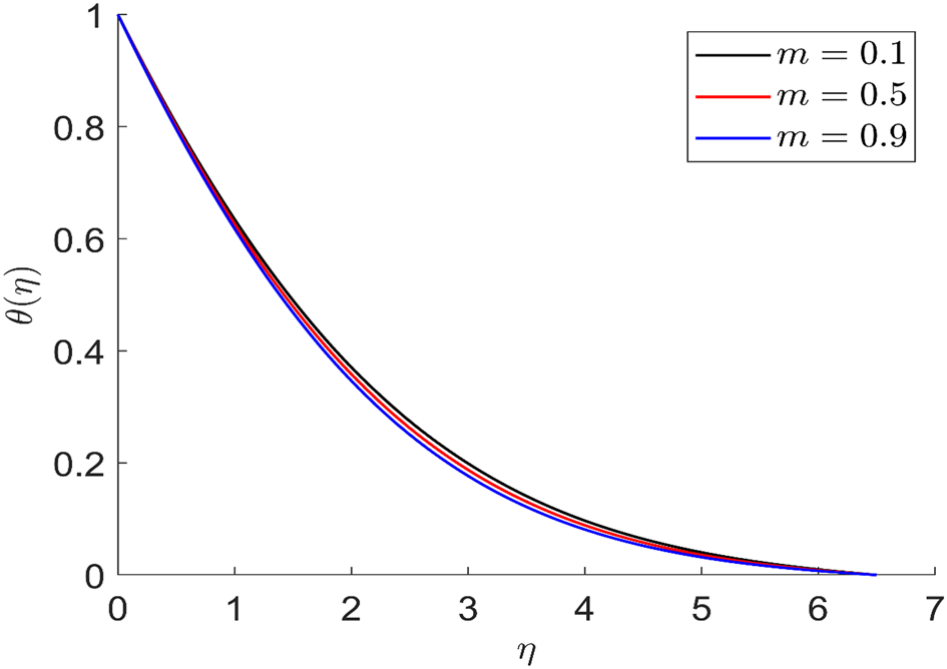
Representation of temperature $\theta \left( \eta \right)$ for the different values of $m$ with R = 3, n = 3, v = 0.1, $\phi$ = 0.2, Pr = 0.6, $\phi_{1}$ = 0.4, $\phi_{2}$ = 0.8, $\beta_{3}$ = 0.5, $\beta$ = 0.4, $\beta_{1}$ = 0.5, $\beta_{2}$ = 0.9, u = 0.2, $\xi$ = 0.2.

**Figure 9:**
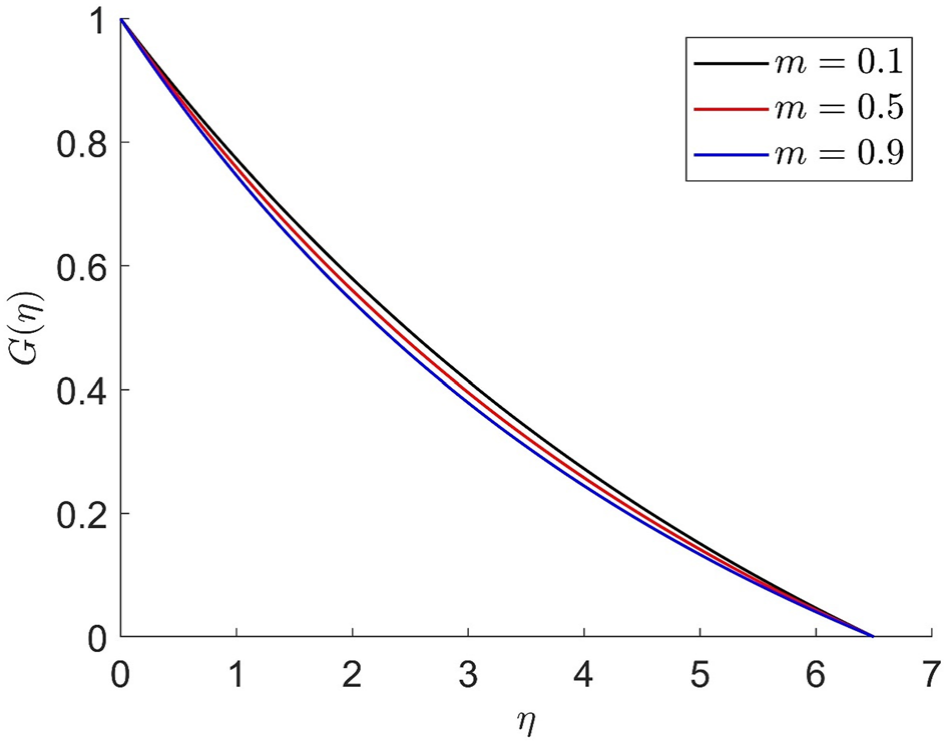
Representation of tangential velocity $g\left( \eta \right)$ for the different values of $m$ with R = 3, n = 3, v = 0.1, $\phi$ = 0.2, Pr = 0.6, $\phi_{1}$ = 0.4, $\phi_{2}$ = 0.8, $\beta_{3}$ = 0.5, $\beta$ = 0.4, $\beta_{1}$ = 0.5, $\beta_{2}$ = 0.9, u = 0.2, $\xi$ = 0.2.

For different values of volume concentration $\left( \varphi \right)$, dimensionless magnetic field intensity $\left( \xi \right)$, dimensionless frequency $\left( {\omega_{0} \tau_{B} } \right)$, Prandtl number $\left( {Pr} \right)$ and ferromagnetic interaction numbers $\left( {\beta ,\beta_{1} ,\beta_{2} ,\beta_{3} } \right)$, a graphical result for axial velocity $\left( f \right)$, radial velocity $\left( {f^{\prime}} \right)$, tangential velocity (g) and temperature $\left( \theta \right)$ has been presented in this work. The BVP4c method in the MATLAB programmer is used to achieve the numerical solution of nonlinear coupled differential equations. The current numerical work is confirmed with prior work after reducing specific physical factors. The dimensionless ferromagnetic interaction numbers $\left( {\beta ,\beta_{1} ,\beta_{2} ,\beta_{3} } \right)$ determine the different types of velocities such as axial velocity, tangential velocity, and radial velocity, as well as temperature distribution. Ethylene glycol is used as the basic fluid in this experiment. The nanoparticles of alumina ${\rm Al}_{2}{\rm O}_{3}$ and Cu are utilized in the preparation. To keep the nanoparticles from clumping together in the transported liquid, ferrofluid was utilized. Table 1 lists the thermophysical characteristics taken into account in this physical model. Figure 2 shows that the axial velocity due to fluctuation of ferromagnetic interaction numbers $\beta$. The current figure physically shows that the fluid becomes more viscous with rising values of $\beta$, which causes the fluid's velocities to decrease. Figures 3, 4, 5 represents the temperature profile for different values of ferromagnetic dimensionless interaction numbers. In this case when increasing the value of ferromagnetic interaction numbers then decrease the temperature profile in the flow field. Figures 6, 7, 8, 9 illustrate that the axial velocity, tangential velocity, temperature profile and radial velocity decrease when increasing the value of dimensionless parameters $m$.

**Figure 10:**
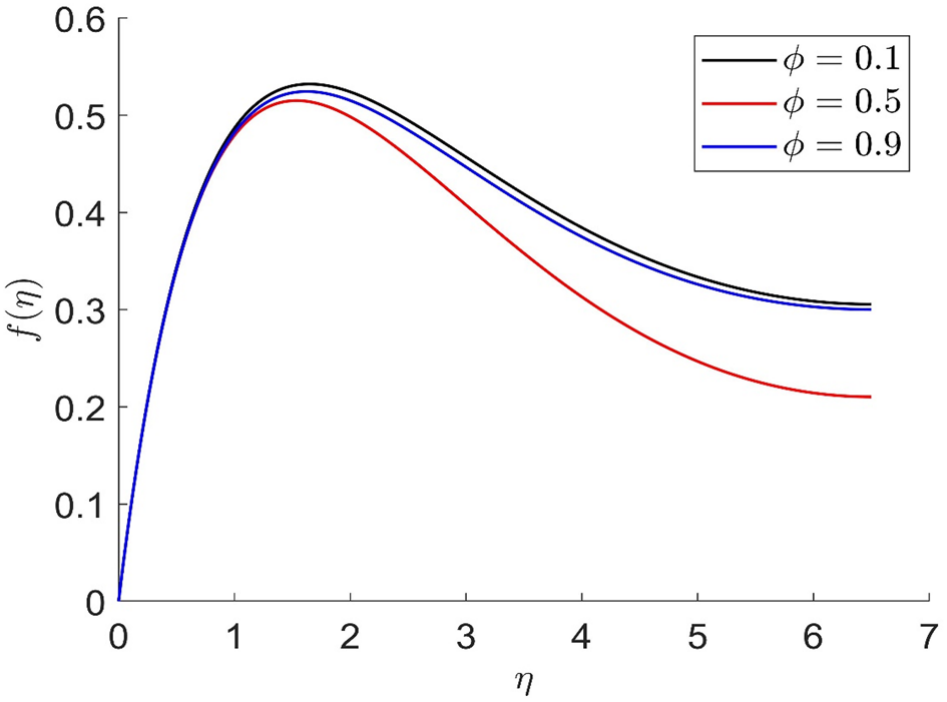
Representation of axial velocity $f\left( \eta \right)$ for the different values of $\phi$ with R = 3, n = 3, v = 0.8, $m$ = 3.5, Pr = 0.7, $\phi_{1}$ = 0.2, $\phi_{2}$ = 0.4, $\beta_{3}$ = 0.6, $\beta$ = 0.4, $\beta_{1}$ = 0.3, $\beta_{2}$ = 0.5, u = 0.4, $\xi$ = 0.4.

**Figure 11:**
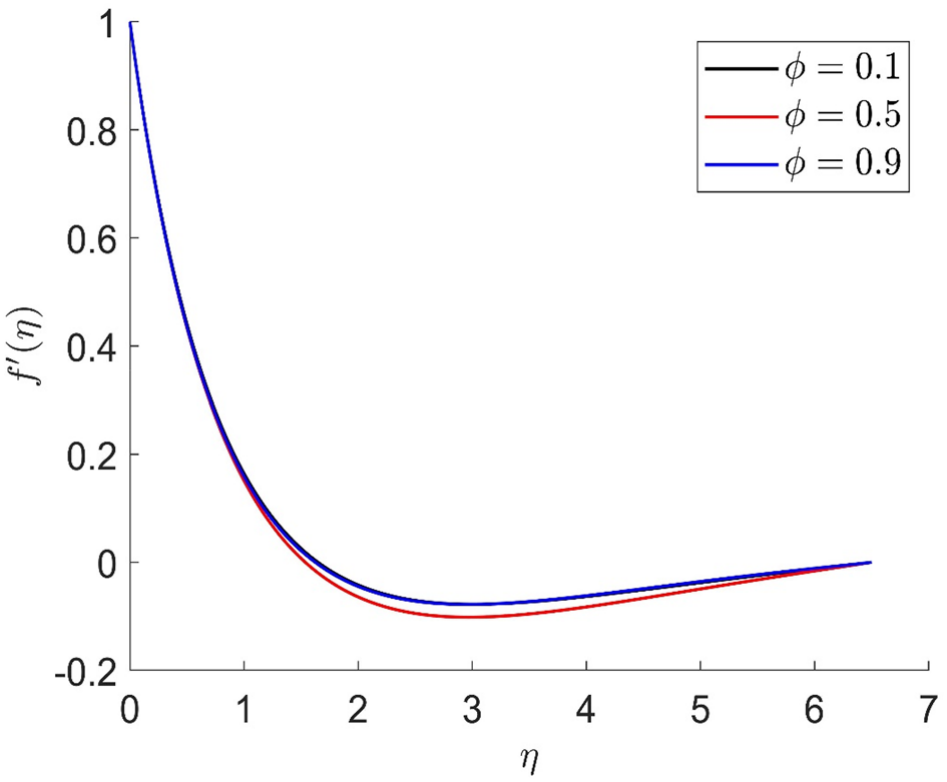
Representation of radial velocity $f^{\prime}\left( \eta \right)$ for the different values of $\phi$ with R = 3, n = 3, v = 0.8, $m$ = 3.5, Pr = 0.7, $\phi_{1}$ = 0.2, $\phi_{2}$ = 0.4, $\beta_{3}$ = 0.6, $\beta$ = 0.4, $\beta_{1}$ = 0.3, $\beta_{2}$ = 0.5, u = 0.4, $\xi$ = 0.4.

**Figure 12:**
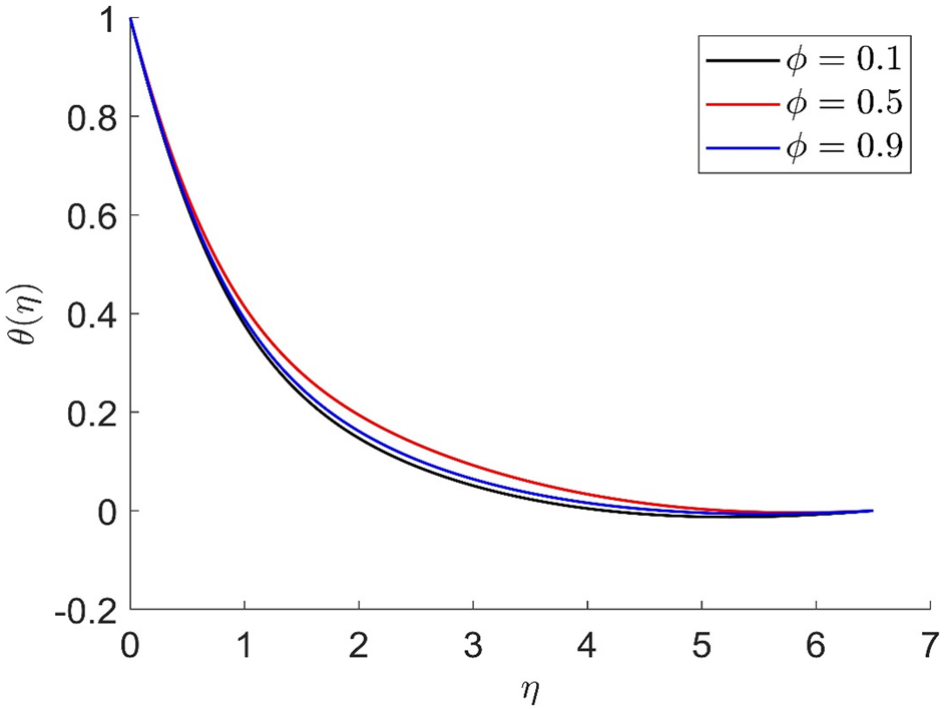
Representation of temperature $\theta \left( \eta \right)$ for the different values of $\phi$ with R = 3, n = 3, v = 0.8, $m$ = 3.5, Pr = 0.7, $\phi_{1}$ = 0.2, $\phi_{2}$ = 0.4, $\beta_{3}$ = 0.6, $\beta$ = 0.4, $\beta_{1}$ = 0.3, $\beta_{2}$ = 0.5, u = 0.4, $\xi$ = 0.4.

**Figure 13:**
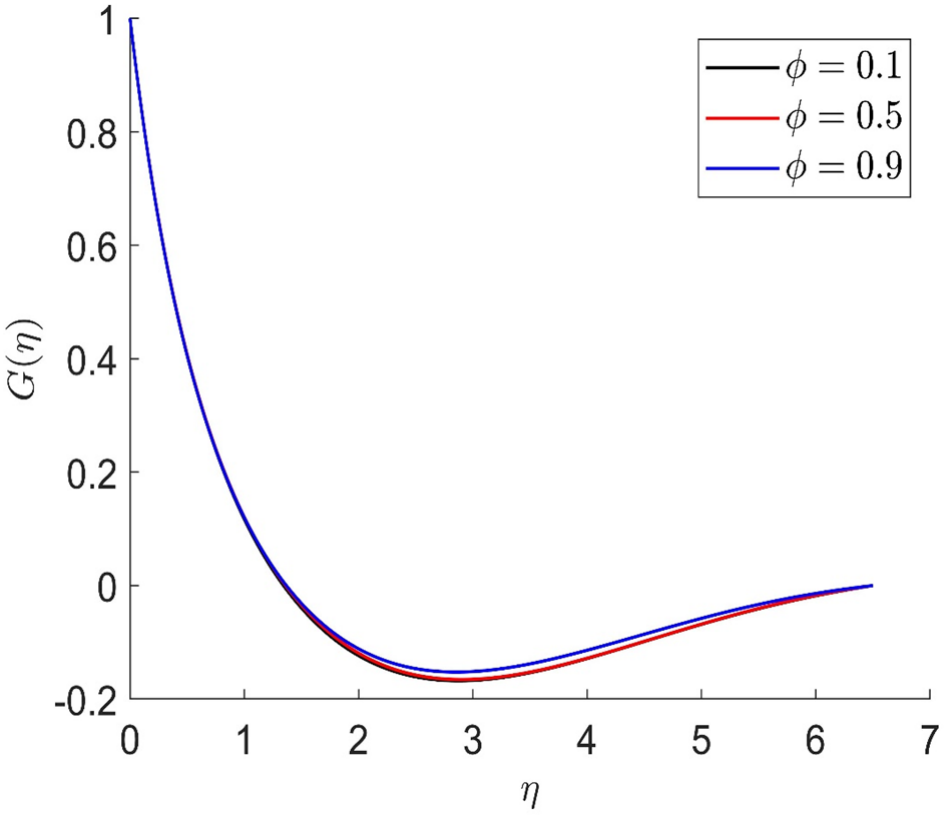
Representation of tangential velocity $g\left( \eta \right)$ for the different values of $\phi$ with R = 3, n = 3, v = 0.8, $m$ = 3.5, Pr = 0.7, $\phi_{1}$ = 0.2, $\phi_{2}$ = 0.4, $\beta_{3}$ = 0.6, $\beta$ = 0.4, $\beta_{1}$ = 0.3, $\beta_{2}$ = 0.5, u = 0.4, $\xi$ = 0.4.

Figures 10, 11, 12, 13 demonstrated the distributions of axial velocity, radial velocity, tangential velocity and temperature profile for distinct values of $\phi$. In this case when $\phi = 0$ then the flow of carried liquids only. If we increase the value of $\phi$ the axial and radial velocity are increased and tangential velocity and temperature are decreased. Volume concentration profile creates the resistance in the flow field, in the existence of magnetic field. The heat transmission in the fluid is improved when the carrier liquid has a greater volume concentration.

**Figure 14:**
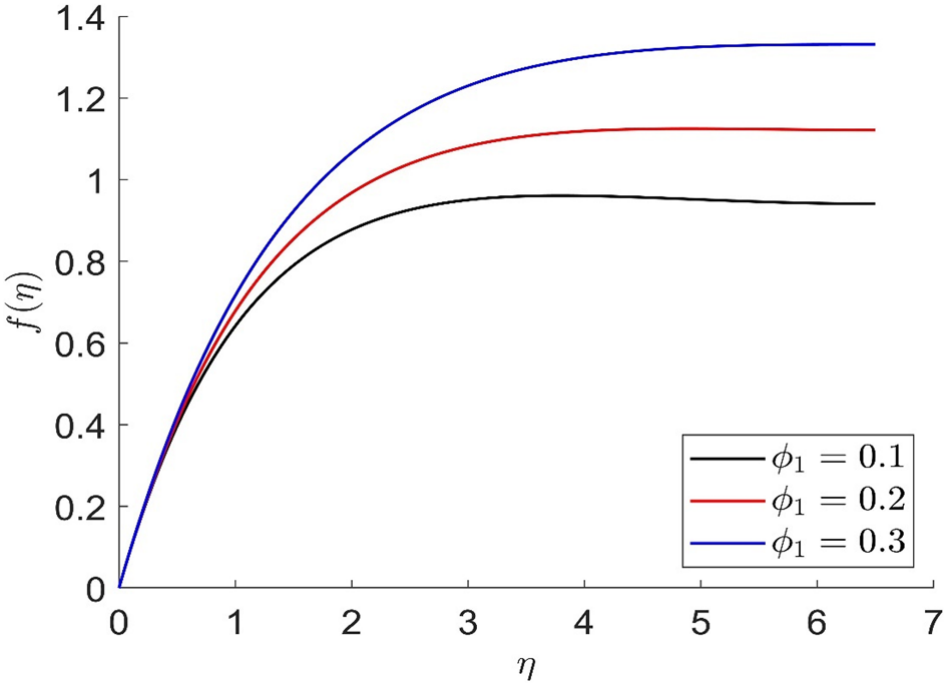
Representation of axial velocity $f\left( \eta \right)$ for the different values of $\phi_{1}$ with R = 3, n = 3, v = 0.8, $m$ = 3.5, Pr = 0.9, $\phi$ = 0.1, $\phi_{2}$ = 0.7, $\beta_{3}$ = 0.6, $\beta$ = 0.3, $\beta_{1}$ = 0.5, $\beta_{2}$ = 0.8, u = 0.5, $\xi$ = 2.5.

**Figure 15:**
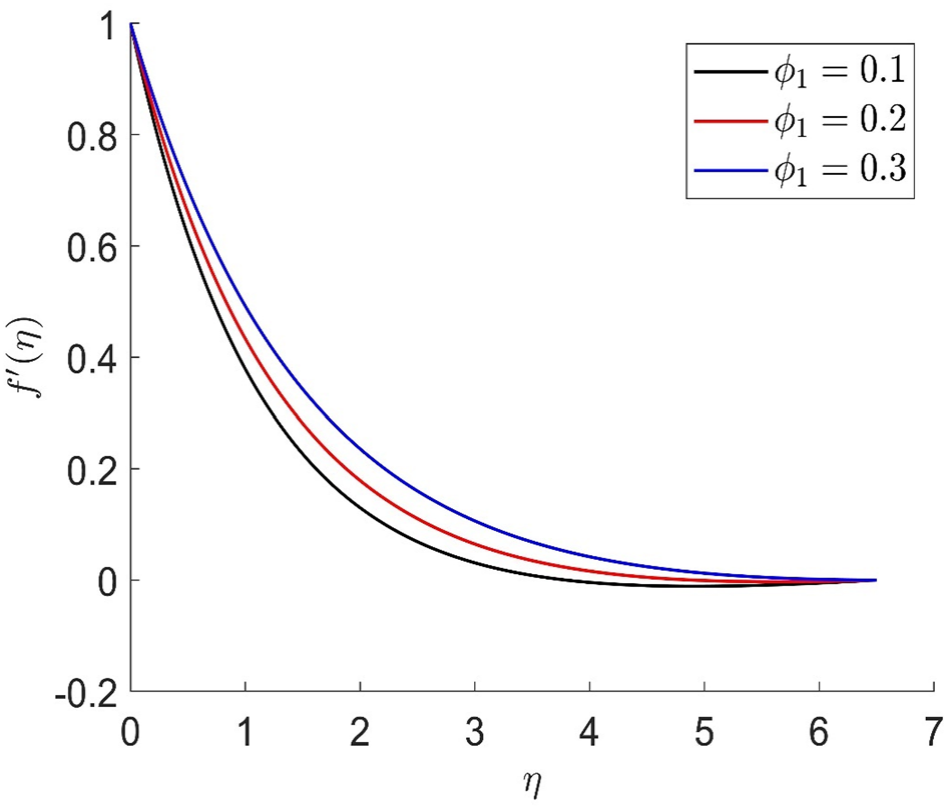
Representation of radial velocity $f^{\prime}\left( \eta \right)$ for the different values of $\phi_{1}$ with R = 3, n = 3, v = 0.8, $m$ = 3.5, Pr = 0.9, $\phi$ = 0.1, $\phi_{2}$ = 0.7, $\beta_{3}$ = 0.6, $\beta$ = 0.3, $\beta_{1}$ = 0.5, $\beta_{2}$ = 0.8, u = 0.5, $\xi$ = 2.5.

**Figure 16:**
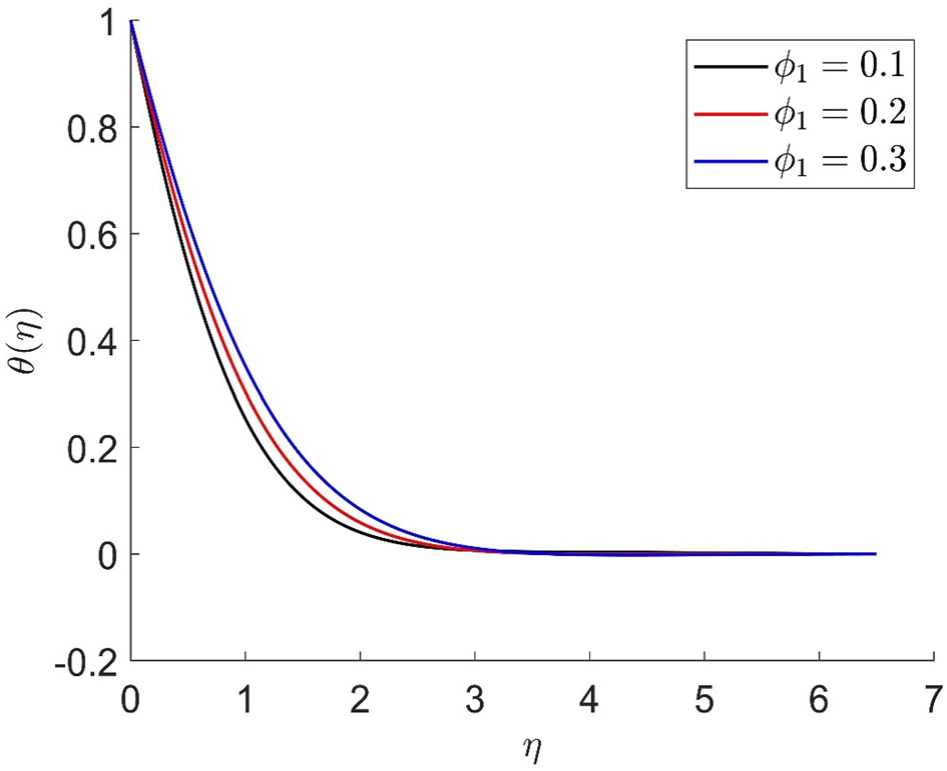
Representation of temperature $\theta \left( \eta \right)$ for the different values of $\phi_{1}$ with R = 3, n = 3, v = 0.8, $m$ = 3.5, Pr = 0.9, $\phi$ = 0.1, $\phi_{2}$ = 0.7, $\beta_{3}$ = 0.6, $\beta$ = 0.3, $\beta_{1}$ = 0.5, $\beta_{2}$ = 0.8, u = 0.5, $\xi$ = 2.5.

**Figure 17:**
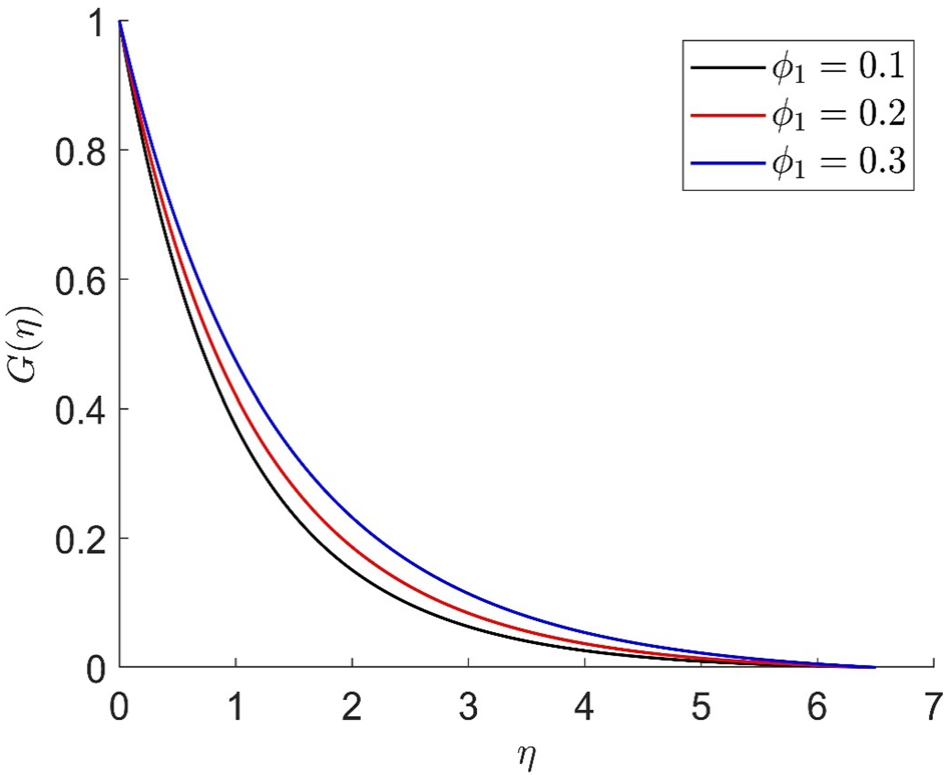
Representation of tangential velocity $g\left( \eta \right)$ for the different values of $\phi_{1}$ with R = 3, n = 3, v = 0.8, $m$ = 3.5, Pr = 0.9, $\phi$ = 0.1, $\phi_{2}$ = 0.7, $\beta_{3}$ = 0.6, $\beta$ = 0.3, $\beta_{1}$ = 0.5, $\beta_{2}$ = 0.8, u = 0.5, $\xi$ = 2.5.

**Figure 18:**
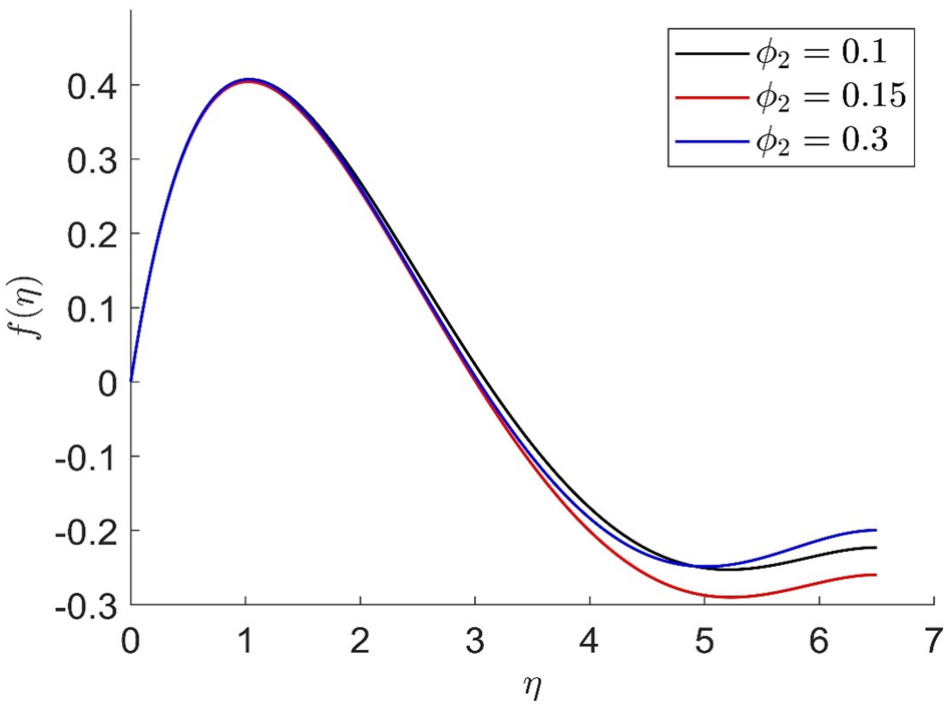
Representation of axial velocity $f\left( \eta \right)$ for the different values of $\phi_{2}$ with R = 7, n = 3, v = 0.2, $m$ = 1.5, Pr = 0.9, $\phi$ = 0.1, $\phi_{1}$ = 0.2, $\beta_{3}$ = 0.9, $\beta$ = 0.4, $\beta_{1}$ = 0.2, $\beta_{2}$ = 0.5, u = 0.3, $\xi$ = 0.2.

**Figure 19:**
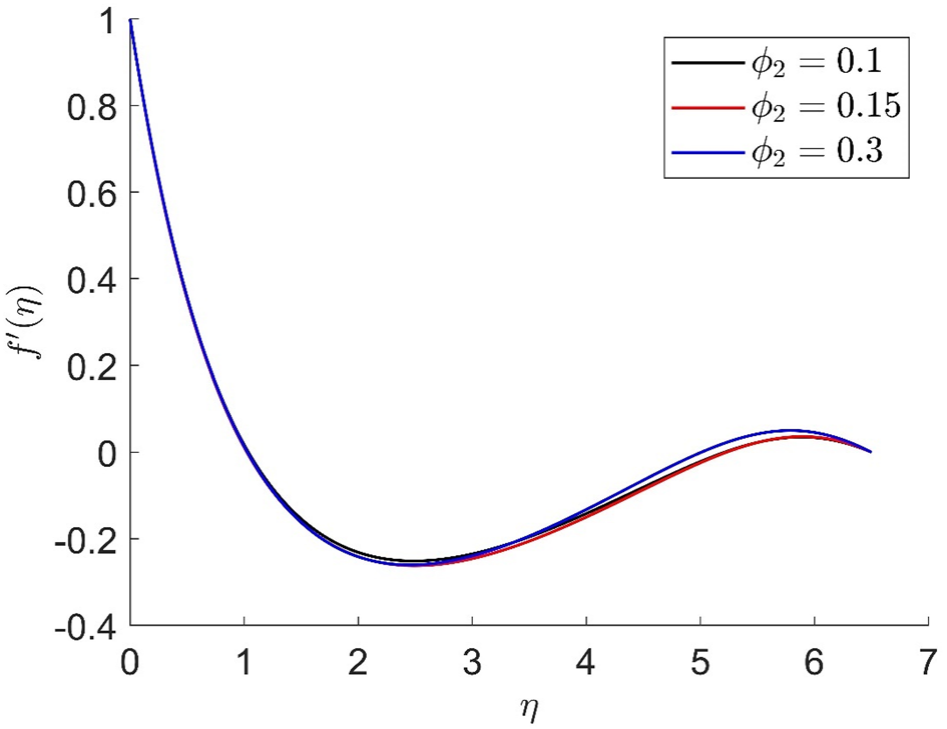
Representation of radial velocity $f^{\prime}\left( \eta \right)$ for the different values of $\phi_{2}$ with R = 7, n = 3, v = 0.2, $m$ = 1.5, Pr = 0.9, $\phi$ = 0.1, $\phi_{1}$ = 0.2, $\beta_{3}$ = 0.9, $\beta$ = 0.4, $\beta_{1}$ = 0.2, $\beta_{2}$ = 0.5, u = 0.3, $\xi$ = 0.2.

**Figure 20:**
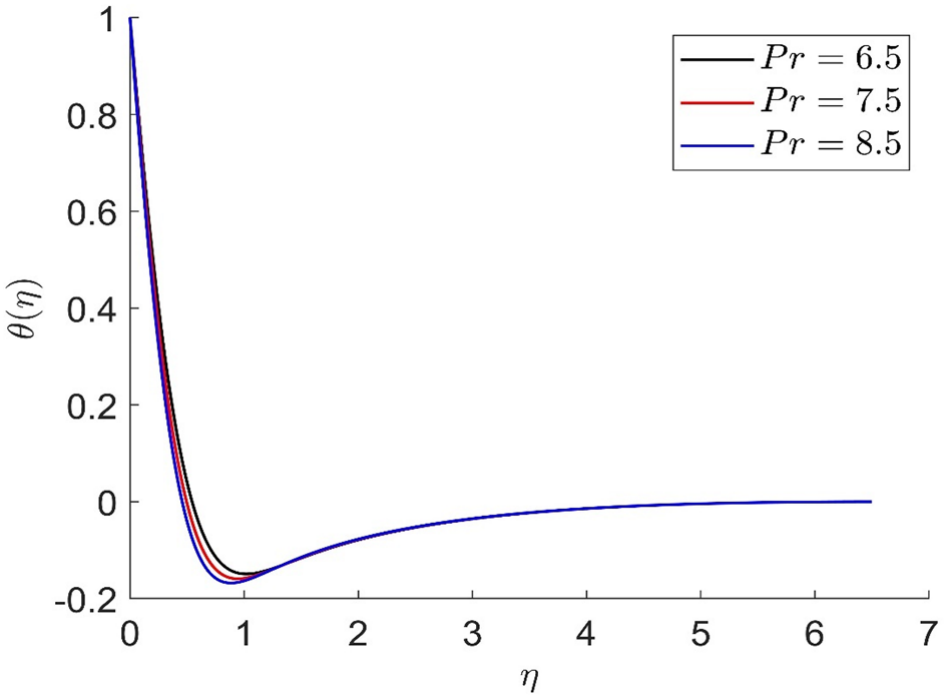
Representation of temperature $\theta \left( \eta \right)$ for the different values of Pr with R = 3, n = 3, v = 0.7, $m$ = 1.5, $\phi_{2}$ = 0.8, $\phi$ = 0.3, $\phi_{1}$ = 0.1, $\beta_{3}$ = 0.5, $\beta$ = 0.4, $\beta_{1}$ = 0.5, $\beta_{2}$ = 0.9, u = 0.2, $\xi$ = 0.2.

In similar manners to Figs. 14, 15, 16, 17, the axial velocity, radial velocity, tangential velocity and temperature are increased when increasing the value of volume concentration $\phi_{1}$. Figures 18 and 19 represent the axial and radial velocity profiles increase when increasing the value of volume concentration $\phi_{2}$. Figures 14, 15, 16, 17, 18, 19) show, the effects of the solid volume fractions of Alumina/Aluminum oxide and Copper/Copper on the thermal field. The volume fraction of Alumina/Aluminium oxide and Cuprum/Copper are boosting the thermal phenomena. However, compared $\phi_{1}$, the thermal profiles in the case of $\phi_{2}$ are more obvious. Due to the nanoparticle volume fractions, the behaviour of these figures is consistent with the physical behaviour of the nanofluid. The thermal conductivity of the nanoparticles is greater than that of the base fluid, which increases the total thermal conductivity of the nanofluid and contributes to the rise in boundary layer temperature. Figure 20 illustrate the temperature profile with the variation of the Prandtl number. The temperature profile is decreased as well as we increase the value of the Prandtl number. This is because the thermal diffusivity of the fluid decreases due to higher values of Pr which further led to the reduction in the thermal boundary layer thickness.

**Figure 21:**
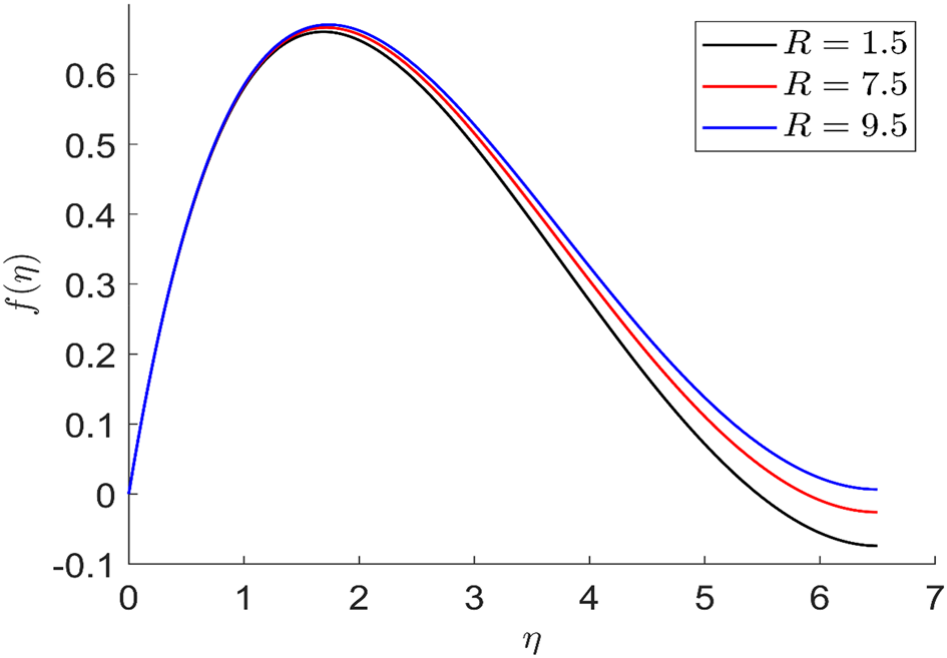
Representation of axial velocity $f\left( \eta \right)$ for the different values of $R$ with R = 3, n = 3, v = 0.7, $m$ = 1.5, $\phi_{2}$ = 0.8, $\phi$ = 0.3, $\phi_{1}$ = 0.1, $\beta_{3}$ = 0.5, $\beta$ = 0.4, $\beta_{1}$ = 0.5, $\beta_{2}$ = 0.9, u = 0.2, $\xi$ = 0.2.

**Figure 22:**
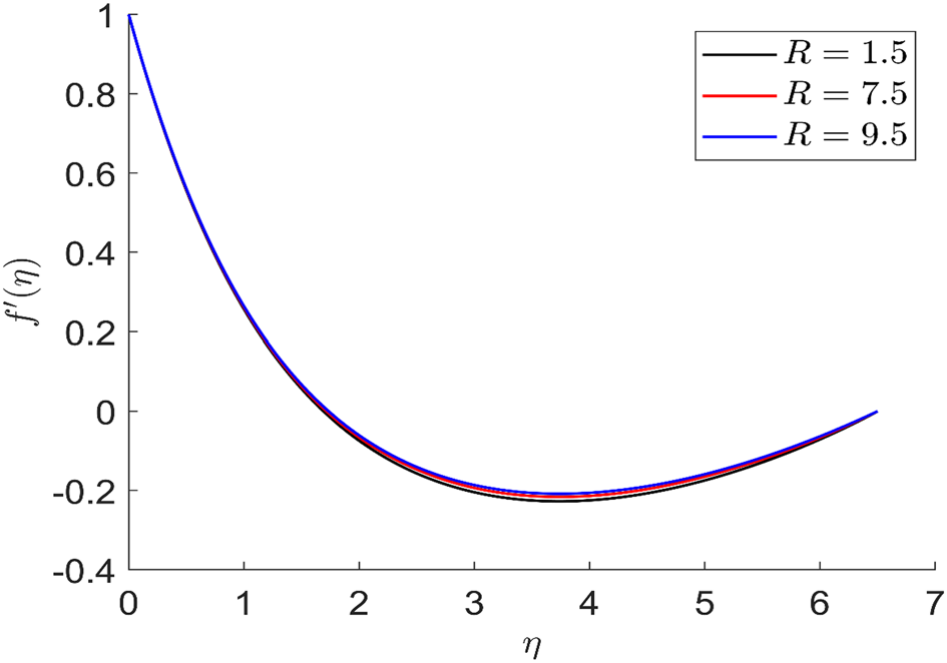
Representation of radial velocity $f^{\prime}\left( \eta \right)$ for the different values of $R$ with R = 3, n = 3, v = 0.7, $m$ = 1.5, $\phi_{2}$ = 0.8, $\phi$ = 0.3, $\phi_{1}$ = 0.1, $\beta_{3}$ = 0.5, $\beta$ = 0.4, $\beta_{1}$ = 0.5, $\beta_{2}$ = 0.9, u = 0.2, $\xi$ = 0.2.

Figures 21 and 22 depict the behavior of axial and radial velocity for different values of the radiative parameter $R$. The axial and radial velocity increased when the value of the radiative parameter $R$ are raised. More heat is introduced to the thermal phenomena as a result of changing the radiation parameter. More heat is introduced to the thermal phenomena as a result of changing the radiation parameter. As a result, the temperature curves are increased by the increasing radiation parameter value. Physically, by raising the value of the parameter R, we may enhance the radiative heat transfer.

**Figure 23:**
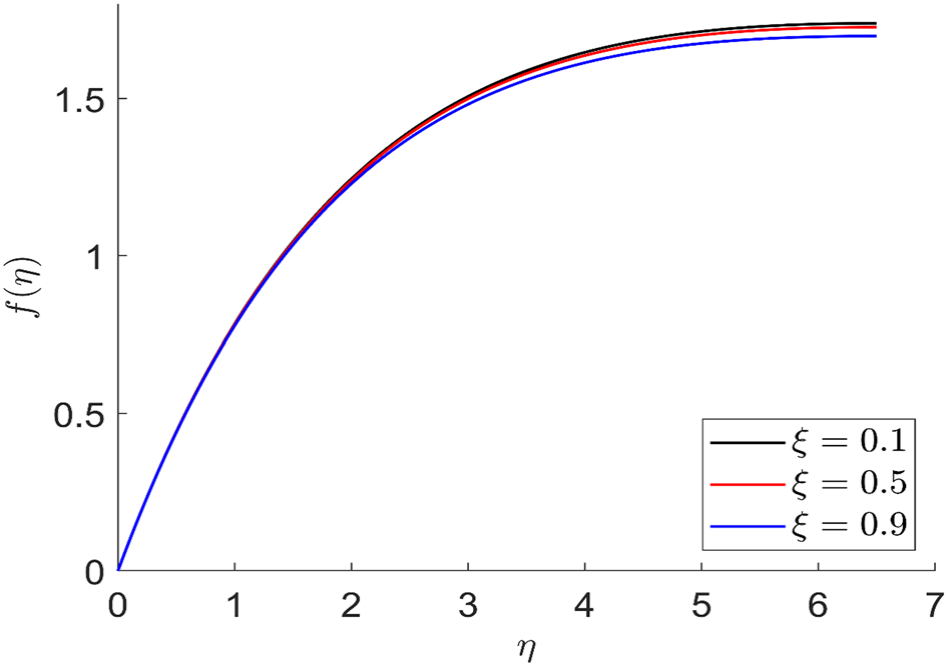
Representation of axial velocity $f\left( \eta \right)$ for the different values of $\xi$ with R = 3, n = 3, u = 0.2, $m$ = 2.5, $\phi_{2}$ = 0.8, $\phi$ = 0.3, $\phi_{1}$ = 0.1, $\beta_{3}$ = 0.5, $\beta$ = 0.4, $\beta_{1}$ = 0.5, $\beta_{2}$ = 0.9, Pr = 0.6, $v$ = 0.1.

**Figure 24:**
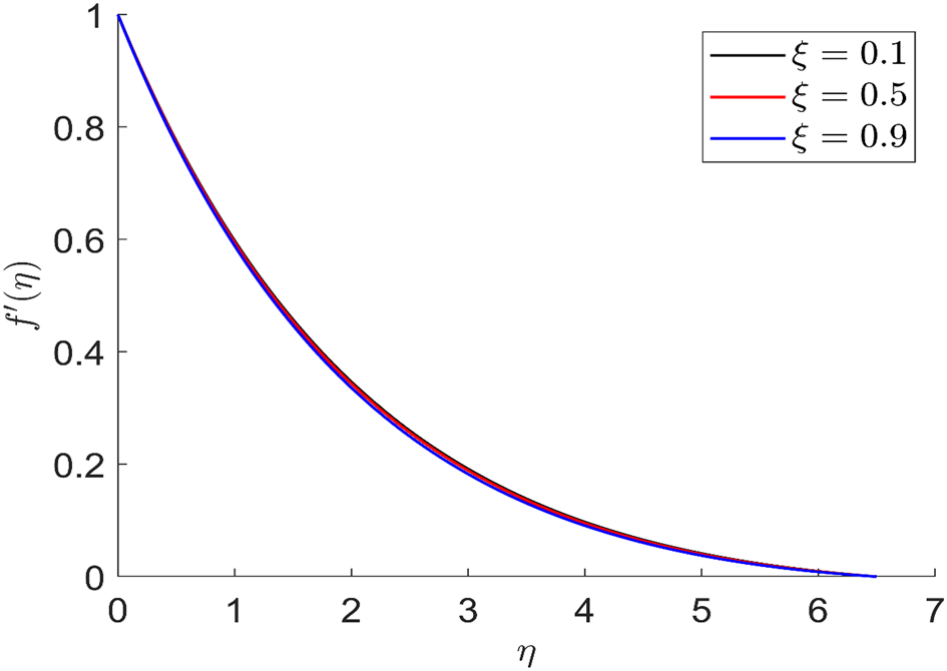
Representation of radial velocity $f^{\prime}\left( \eta \right)$ for the different values of $\xi$ with R = 3, n = 3, u = 0.2, $m$ = 2.5, $\phi_{2}$ = 0.8, $\phi$ = 0.3, $\phi_{1}$ = 0.1, $\beta_{3}$ = 0.5, $\beta$ = 0.4, $\beta_{1}$ = 0.5, $\beta_{2}$ = 0.9, Pr = 0.6, $v$ = 0.1.

Figures 23 and 24 represent the distributions of axial and radial velocity for different values of dimensionless magnetic field intensity $\left( \xi \right)$. When the value of the dimensionless magnetic field is increased, the axial and radial velocity distributions are reduced. As greater resistance to the flow phenomena is created by the application of the magnetic field, the velocity field also decreases. Therefore, a decrease in the velocity curves $f\left( \eta \right)$ and $f^{\prime}\left( \eta \right)$ is observed as a result of an improvement in the magnetic field intensity $\left( \xi \right)$. The various axial and radial velocity profiles all meet their respective boundary criteria.

In Table 2 for distinct values of volume concentration $\left( {\phi ,\phi_{1} ,\phi_{2} } \right)$, the heat transfer increases. Whereas ferromagnetic interaction numbers $\left( {\beta ,\beta_{1} ,\beta_{2} ,\beta_{3} } \right)$ is the opposite. The heat transfer increases as dimensionless magnetic field intensity $\xi$ is increased. The heat transmission in the fluid is reduced as the Prandtl number is increased. Table 3. demonstrate that the findings are in perfect accord with the results of the literature (Turkyilmazoglu^77^, Hafeez et al.^49^).

**Table 2:** Variation of $$Nu\left( {{\text{Re}}_{x} } \right)^{{ - \left( {{\raise0.7ex\hbox{$1$} \!\mathord{\left/ {\vphantom {1 2}}\right.\kern-\nulldelimiterspace} \!\lower0.7ex\hbox{$2$}}} \right)}}$$ for distinct number of parameters.

$$\phi$$	$$\phi_{1}$$	$$\phi_{2}$$	$$\beta$$	$$\beta_{1}$$	$$\beta_{2}$$	$$\beta_{3}$$	$$\omega_{0}$$	$$\tau_{B}$$	$$m$$	$$R$$	$$\Pr$$	$$\xi$$	$$Nu\left( {{\text{Re}}_{x} } \right)^{{ - \left( {{\raise0.7ex\hbox{$1$} \!\mathord{\left/ {\vphantom {1 2}}\right.\kern-\nulldelimiterspace} \!\lower0.7ex\hbox{$2$}}} \right)}}$$
0.1	0.2	0.4	0.4	0.3	0.5	0.6	0.4	0.8	3.5	3	6.5	0.4	− 0.003481
0.5													− 0.002873
0.9													− 0.002553
0.1	0.1	0.7	0.3	0.5	0.8	0.6	0.5	0.8	3.5	3	6.9	2.5	0.000182
	0.2												0.000079
	0.3												− 0.000018
0.1	0.2	0.1	0.4	0.2	0.5	0.9	0.3	0.2	1.5	7	7.9	0.2	− 3.237272
		0.15											0.401806
		0.3											0.389120
0.3	0.1	0.8	0.1	0.2	0.5	0.5	0.2	0.3	1.5	3	8.6	0.2	− 0.000146
			0.4										− 0.000122
			0.9										− 0.000072
0.3	0.1	0.8	0.4	0.1	0.5	0.5	0.2	0.3	1.5	3	7.6	0.2	− 0.000119
				0.4									− 0.000178
				0.9									− 0.000277
0.3	0.1	0.8	0.4	0.5	1.5	0.5	0.2	0.7	1.5	3	9.5	0.2	− 0.000157
					3.5								− 0.000157
					5.5								− 0.000157
0.3	0.1	0.8	0.4	0.5	0.9	0.1	0.2	0.7	1.5	3	9.6	0.2	− 0.000093
						0.5							− 0.000155
						0.9							− 0.000217
0.3	0.2	0.3	0.4	0.2	0.5	0.7	0.1	0.3	1.5	3	7.9	0.2	− 0.167494
							0.5						− 0.169079
							0.7						− 0.173604
0.3	0.2	0.3	0.4	0.2	0.5	0.7	0.3	0.1	1.5	3	9.2	0.2	0.293082
								0.5					0.221934
								0.7					0.188009
0.2	0.4	0.8	0.4	0.5	0.9	0.5	0.2	0.1	0.1	3	7.6	0.2	− 0.001668
									0.5				− 0.001287
									0.9				− 0.001019
0.1	0.2	0.5	0.4	0.5	0.6	0.8	0.2	0.9	1.5	1.5	8.3	0.3	− 19.405058
										7.5			− 11.060991
										9.5			− 3.075276
0.3	0.1	0.8	0.4	0.5	0.9	0.5	0.2	0.7	1.5	3	6.6	0.2	− 0.000230
											7.6		− 0.000198
											8.6		− 0.000174
0.3	0.1	0.8	0.4	0.5	0.9	0.5	0.2	0.1	2.5	3	9.6	0.1	− 0.000087
												0.5	− 0.000085
												0.9	− 0.000079

**Table 3:** Comparison of $f^{\prime}(0)$, $f^{\prime\prime}(0)$ and $\theta^{\prime}(0)$ for $\Pr = 6.2,\,$ $\beta = \beta_{1} = \beta_{2} = 0,\,$ $\Phi = 0$.

	Turkyilmazoglu^77^	Hafeez et al.^49^	Present Resul
$f^{\prime}(0)$	0.51023262	0.510 116 26	0.51032163
$f^{\prime\prime}(0)$	0.61592201	0.615 849 27	0.61575468
$\theta^{\prime}(0)$	0.93387794	0.933 694 11	0.93375564

## Conclusion

In this work, the flow and heat transport across a spinning disc under the influence of a nonlinearly extended alternating magnetic field in a radial direction are investigated. For the equations to be self-similar, stretching velocities may be obtained through lie group analysis in two ways: linear and power-law. The governing partial differential equations are turned into a system of coupled ordinary nonlinear differential equations with suitable similarity transformations. The following are the key conclusions from the study:

- When an alternating magnetic field is present, additional flow resistance is formed by the volume concentration and the dimensionless magnetic field intensity. The heat transmission in the fluid is enhanced by rotational viscosity when the magnetic field is stationary, i.e., $\omega_{0} \tau_{B} = 0$. The heat transmission in an alternating magnetic field is determined by the frequency of the field.
- The ferromagnetic interaction numbers are noteworthy in defining the thickness of the momentum and thermal boundary layers. The heat transmission in the fluid is reduced as the Prandtl number is increased.
- The existence of a fixed magnetic field increases the flow resistance to the maximum. When the dimensionless field frequency is unity, the magnetic field does not affect viscosity. The rotational viscosity of ferromagnetic fluid becomes negative when the dimensionless field frequency is larger than one.
- It has been found that hybrid nanofluid flow outperforms nanofluid flow in terms of Nusselt number or heat transfer rate.

In future, similar work can be performed for flow over a surface and in the cylinderical domain.

## List of symbols

$$\rho_{n}$$: Density $${\text{Kg}}/{\text{m}}^{3}$$
$$V$$: Velocity $${\text{m}}/{\text{s}}$$
$$p$$: Pressure $${\text{Kgm}}^{ - 1} {\text{s}}^{ - 2}$$
$$\mu_{n}$$: Reference viscosity $${\text{Pa}}.{\text{s}}$$
$$M$$: Magnetization
$H$: Magnetic field intensity ${\text{W}}/{\text{m}}^{2}$
$\tau_{s}$: Rotational relaxation time
$$\omega_{p}$$: Angular velocity $${\text{m}}/{\text{s}}$$
$$\phi$$: Dissipation function
$$\varphi$$: Volume fraction
$$\psi$$: Tangential axes
$$u$$: Radial direction
$$w$$: Axial direction
$$\Pr$$: Prandtl number $${\text{m}}^{2} /{\text{s}}$$
$$T_{\infty }$$: Ambient temperature $$({\text{K}})$$
$s$: Radial stretching of disk
$\rho_{s}$: Density of solid ${\text{Kg}}/{\text{m}}^{3}$
$\left( {\rho c_{p} } \right)_{nf}$: Heat capacitance of nanofluid ${\text{J}}/{\text{K}}$
$$\Omega$$: Vorticity
$\tau_{B}$: Brownian relaxation time
$M_{0}$: Instantaneous equilibrium magnetization
$I$: The moment of inertia ${\text{Kg}}.{\text{m}}^{2}$
$$t$$: Time $${\text{s}}$$
$$c_{p}$$: Specific heat $${\text{J}}.{\text{Kg}}^{ - 1} .{\text{K}}^{ - 1}$$
$$T$$: Temperature $${\text{K}}$$
$$k_{n}$$: Thermal conductivity $${\text{Wm}}^{ - 1} {\text{K}}^{ - 1}$$
$$B$$: Magnetic induction $${\text{T}}$$
$\xi_{0}$: Strength of the magnetic field ${\text{T}}$
$$r$$: Radial axes
$$v$$: Tangential direction
$${\text{Re}}$$: Reynolds number
$$T_{w}$$: Wall temperature $${\text{K}}$$
$\omega$: Uniform angular velocity ${\text{m}}/{\text{s}}$
$\rho_{f}$: Density of fluid ${\text{Kg}}/{\text{m}}^{3}$
$\mu_{f}$: Viscosity of fluid ${\text{Pa}}.{\text{s}}$
$k_{nf}$: Effective thermal conductivity ${\text{Wm}}^{ - 1} {\text{K}}^{ - 1}$
